# Rendering compact formation and trajectory tracking for cyber unmanned ground vehicles

**DOI:** 10.1016/j.fmre.2023.08.018

**Published:** 2025-03-03

**Authors:** Xiaomin Zhao, Zhengrong Cui, Chee-Meng Chew, Fangfang Dong

**Affiliations:** a School of Automotive and Transportation Engineering, Hefei University of Technology, Hefei 230009, China; b Department of Mechanical Engineering, National University of Singapore, Singapore 117575, Singapore; c School of Mechanical Engineering, Hefei University of Technology, Hefei 230009, China

**Keywords:** Cooperative control, Cyber interference, Swarm system, Unmanned ground vehicle, Constraint

## Abstract

This paper considers the cooperative control problem for the unmanned ground vehicle swarm system with cyber interference. Each vehicle in the system is intelligent and connected with each other via networks. The cyber security issue is inevitable and vital in the control design for the swarm system. Taking account of the cyber interference and system uncertainty, we explore the cooperative control for the unmanned ground vehicle swarm system. In order to describe the behaviors of mutual attraction, repulsion and overall trajectory tracking in a mathematical manner, we abstract the integrated potential function, thereby the kinematic model. Treating the mathematical model as the system constraint and decomposing the system uncertainty, we propose a class of cooperative adaptive robust controls to assure that all the unmanned ground vehicles follow the constraint to move. Four major system performances are achieved: (i) global stability (i.e., uniform boundedness and uniform ultimate boundedness), (ii) compact formation, (iii) cooperative hunting, (iv) trajectory tracking.

## Graphical abstract

**Figure:**
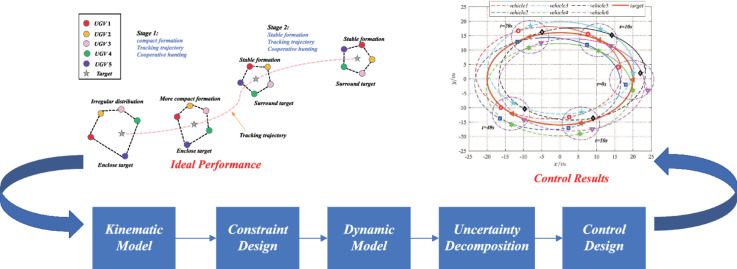

## 1 Introduction

In the biological world, swarm behavior is a common sight, which can be seen everywhere. Examples can be found in certain social species, such as ant colonies, fish schools and sheep flocks. Based on simple interaction and collaboration between each other, these biological swarms can finalize some sophisticated tasks, which may be difficult or even impossible for any single individual to complete [1]. With the advanced technology and deepening research, engineers introduced the concept of swarm into mechanical systems and thereby derived the swarm mechanical systems. Nowadays, swarm mechanical systems are no longer unfamiliar. The research on swarm mechanical systems spans multiple fields and disciplines, such as unmanned aerial vehicle (UAV) swarm systems, unmanned ground vehicle swarm systems and unmanned underwater vehicle swarm systems [2], [3], [4]. Each agent in a swarm mechanical system is erected by mechanical devices. They follow the similar pattern as in biological swarms and possess a wide range of potential applications.

Mimicking the behaviors of social animals, a swarm of coordinated robotic agents can be used for shepherding [5], military security affairs [6], disaster relief and rescue work [7]. In the biomedical field, the magnetic microrobotic swarms with highly concentrated nanopreparations are expected to be applied for targeted drug delivery tasks [8], [9]. The increasing number of vehicles leads to the traffic jam problem. Hence, the Intelligent Transportation Systems (ITS) have been brought up. In the field of marine science, autonomous underwater vehicle (AUV) swarms are helpful for marine resource development and underwater target tracking, which can also be used as a network basis to build ITS for marine pollution monitoring [10]. As an important part and new trend of ITS, the Internet of Vehicles (IOVs) have attracted extensive attention of scientific and industrial circles. The essence of IOV is the application of Internet of Things (IOT) in ITS. IOVs make the application of swarm concept in ITS workable. The introduction of swarm architecture makes the ITS more compact, safe, economical and efficient [11]. A holistic framework for fully autonomous ground vehicles (FAGVs) in the smart city is proposed in [12], in which the swarm behavior coordination of FAGVs contributes to the construction of a safer, sustainable and efficient smart city.

Herd animals rely on chemicals or signs to communicate and coordinate their corresponding synergistic tasks, while the information interaction between agents in the artificial swarm system relies on various communication networks [13]. The individual agents should have capabilities of communication, sensing and local processing [14]. From this perspective, the artificial swarm systems can also be treated as cyber-physical systems (CPSs) in a sense [15]. When the system is interfered by network, catastrophic or even fatal consequences may be brought about if there are no proper defensive countermeasures. Therefore, the cyber interference issues are vitally important as a branch of network security in the swarm mechanical systems. In this context, consideration of the network and cyber space is a prerequisite for the proper control of artificial swarms. In order to compensate for interference lost and guarantee the system performance, effective resilient control strategies also received significant attention in the academic research. In [16], a distributed output-feedback controller is proposed to handle the cooperative output containment problem of the multigroup systems under cyber interference and infiltration. A decentralized hybrid controller targeting homogeneous vehicle platoons is designed in [17], which can show an effective resilience to DoS attacks and guarantee the system string stability. In addition, event-triggered mechanism [18], model predictive control methods [19], adaptive sliding-mode cooperative control [20] and robust control theories [21] have also been demonstrated valid to enhance the cyber security and robustness of the CPSs.

This paper explores the cooperative control problem for the unmanned ground vehicle (UGV) swarm system with system uncertainty and cyber interference. The main contributions of this paper can be summarised as follows. (i) The integrated kinematic model including compact formation, cooperative hunting and trajectory tracking of the UGV swarm system is presented, which is further regarded as the active constraints. (ii) Considering both system uncertainty and cyber interference inputs, the dynamic model of the UGV swarm system is constructed based on the proposed kinematic model. (iii) On the premise of decomposition of uncertainty and cyber interference input, a novel adaptive robust controller is proposed. The resilience to cyber interference is demonstrated by both theoretical derivation and simulations. The cooperative control drives the UGVs to achieve the kinematic performances approximately. We can state this paper as follows. Section 2 depicts the ideal kinematic model for the UGV swarm system. Section 3 presents the dynamic model for the swarm system under cyber interference. Section 4 articulates the design process of the cooperative control. Section 5 analyses the control performance of the system. Section 6 raises an illustrative example. Section 7 presents the final conclusions.

## 2 The integrated kinematic model

We consider an UGV swarm system composed of n agents, wherein the position information of all agents in the swarm system is accessible. We use $p^{i}\left(t\right)\in R^{k}$ to denote the position vector of the unmanned ground vehicle i, $i\in N^{+}$, $N^{+}=\left\{1,2,\cdots ,n\right\}$. Taking the performances of compact formation, cooperative hunting and trajectory tracking into account, an integrated type of kinematic model for the ith UGV is presented as

(1) $${\dot{p}}^{i}=-\sum_{j=1,j\neq i}^{n}\frac{\partial U_{ij}}{\partial p^{i}}\left(p^{i},p^{j}\right)-\frac{\partial U_{i*}}{\partial p^{i}}\left(p^{i},p^{*}\right)+{\dot{p}}^{*},$$

here $i,j\in N^{+}$, $U_{ij}\left(\cdot \right)$ represents the potential function between UGV i and UGV j, $U_{i*}\left(\cdot \right)$ represents the potential function between UGV i and the reference trajectory (or the target). The first term on the right hand of (1) represents the performance of compact formation, while the second term represents the trajectory tracking performance. Both the first term and second term contributes to the performance of cooperative hunting.

**Fig. 1:**
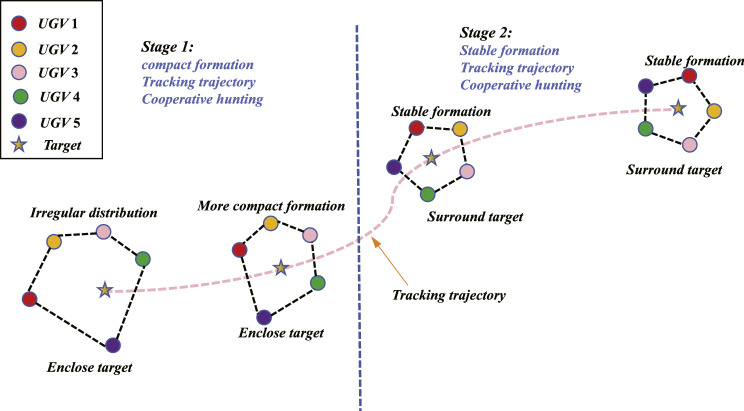
**The desirable motion diagram of the UGV swarm (**n=5**)**.

Take n=5 for example, the desirable motion diagram of the UGV swarm is shown in Fig. 1. That means, in the desired state, the target is always surrounded by the n UGVs, meanwhile, these UGVs can construct a stable formation and track the reference trajectory.

Let $u_{ij}=\partial U_{ij}\left(p^{i},p^{j}\right)/\partial p^{i}$ denote the attraction/repulsion function. We define $\bar{p}=\left(1/n\right)\sum_{i=1}^{n}p^{i}$ as the swarm centroid. Let $p^{*}\left(t\right)\in R^{k}$ represent the reference trajectory of $\bar{p}$, then we define $u_{i*}=\partial U_{i*}\left(p^{i},p^{*}\right)/\partial p^{i}$. Serving for the kinematic model, the following properties need to be satisfied [22], [23].

Property 1 *The potential function* $U_{ij}\left(p^{i},p^{j}\right)$ *can be converted to a new function* ${\tilde{U}}_{ij}\left(\cdot \right)$*:* $R^{k}\to R$ *with respect to the relative position vector* $\left(p^{i}-p^{j}\right)$*. That is,*

(2) $$\begin{array}{c} U_{ij}\left(p^{i},p^{j}\right)={\tilde{U}}_{ij}\left(p^{i}-p^{j}\right). \end{array}$$

*Similarly, there exists a new function* ${\tilde{U}}_{i*}\left(\cdot \right)$ *that guarantees*

(3) $$\begin{array}{c} U_{i*}\left(p^{i},p^{*}\right)={\tilde{U}}_{i*}\left(p^{i}-p^{*}\right). \end{array}$$

Property 2 *For each* $i,j\in N^{+}$ *and* i≠j*, the following functions have symmetric characteristics:*

(4) $$U_{ij}(\left(p^{i},p^{j}\right)=U_{ji}(\left(p^{j},p^{i}\right).$$

(5) $$U_{i*}(\left(p^{i},p^{*}\right)=U_{*i}(\left(p^{*},p^{i}\right).$$

Property 3 *For each* $i,j\in N^{+}$ *and* i≠j*, there exists a hypersphere* $O_{ij}\left(p^{i}\right)$ *with center* $p^{i}$ *and radius* $r_{ij}$*, such that (i) if* $p^{j}\in O_{ij}\left(p^{i}\right)$*, then* ${\left(p^{i}-p^{j}\right)}^{T}u_{ij}\left(p^{i},p^{j}\right)<0$*; (ii) if* $p^{j}\notin O_{ij}\left(p^{i}\right)$*, then* ${\left(p^{i}-p^{j}\right)}^{T}u_{ij}\left(p^{i},p^{j}\right)>0$*.*

Property 4 *For the function* $u_{ij}$*, there exists a new function* ${\bar{u}}_{ij}\left(p^{i},p^{j}\right)$*:* $R^{k}\times R^{k}\to R$*, such that*

(6) $$\begin{array}{c} u_{ij}\left(p^{i},p^{j}\right)=\left(p^{i}-p^{j}\right){\bar{u}}_{ij}\left(p^{i},p^{j}\right). \end{array}$$

*Further, we can get*

(7) $$\begin{array}{c} ∥\left(p^{i}-p^{j}\right)\left({\bar{u}}_{ij}\left(p^{i},p^{j}\right)-a_{ij}\right)\left|\right|\leq \psi_{ij}, \end{array}$$

*where* $a_{ij}$ *and* $\psi_{ij}$ *are positive scalar constants.*

Property 5 *The function* $u_{i*}$ *can be expressed as*

(8) $$\begin{array}{c} u_{i*}\left(p^{i},p^{*}\right)=\left(p^{i}-p^{*}\right){\bar{u}}_{i*}\left(∥p^{i}-p^{*}∥\right). \end{array}$$

*For simplicity, let* $p^{i*}:=p^{i}-p^{*}$, and the function ${\bar{u}}_{i*}\left(\cdot \right)$ *will satisfy that*

(9) $$\left\{ \begin{array}{cc} {\bar{u}}_{i*}(∥p^{i*}∥)>0, & \;\;\text{if}\;∥p^{i*}∥>0,\\ {\bar{u}}_{i*}(∥p^{i*}∥)=0, & \;\;\text{if}\;∥p^{i*}∥=0. \end{array}\right.$$

Remark 1 Properties 1 and 2 reveal that the inter-individual action within the swarm only relies on the relative position, and these roles are mutual. Property 3 shows that the direction of the attraction/repulsion function $u_{ij}\left(\cdot \right)$ is determined. The repulsive force is shown when the relative distance is too close, while the attractive force is shown when the relative distance is far enough. Since the equilibrium distance is $r_{ij}$, the collision avoidance can be converted as $r_{ij}>2r_{s}$, where $r_{s}$ is the safety radius. Properties 4 and 5 show the linear factorization of the corresponding functions $u_{ij}\left(\cdot \right)$ and $u_{i*}\left(\cdot \right)$. The inequality 2.7 in Property 4 can also lay the foundation for the realization of the following Performance 2. All of the properties indicate the formation characteristics of the swarm.

Based on the above properties, the following performances will be derived.

Performance 1 For any $i,j\in N^{+}$, we have

(10) $$u_{ij}\left(p^{i},p^{j}\right)=-u_{ji}\left(p^{j},p^{i}\right),$$

(11) $$u_{i*}\left(p^{i},p^{*}\right)=-u_{*i}\left(p^{*},p^{i}\right).$$

Performance 2 Let ${\hat{e}}_{i}:=p^{i}-\bar{p}$, and the error vector $\hat{e}:={\left[{\hat{e}}_{1}^{T},{\hat{e}}_{2}^{T},\cdots ,{\hat{e}}_{N}^{T}\right]}^{T}$. Under the properties 1–5, the error vector is uniformly bounded (For any r>0, there exists a positive d(r)<∞, then $∥\hat{e}\left(t\right)∥\leq d\left(r\right)$ for all $t\geq t_{0}$ if $∥\hat{e}\left(t_{0}\right)∥\leq r$ is met) and uniformly ultimately bounded (For any r>0 and $∥\hat{e}\left(t_{0}\right)∥\leq r$, there is a $\underset{‾}{d}>0$ and a positive $T(\bar{d},r)<\infty$ such that $∥\hat{e}\left(t\right)∥\leq \bar{d}$ for any $\bar{d}>\underset{‾}{d}$ as $t\geq t_{0}+T\left(\bar{d},r\right)).$

Performance 3 Let $∥e_{c*}∥=∥\bar{p}-p^{*}∥$ denote the swarm tracking error. Subject to Properties 1–4, $\lim_{t\to \infty }∥e_{c*}\left(t\right)∥=0$. When t→∞, we can get

(12) $$\begin{array}{c} p^{*}\in conv\left\{p^{1},p^{2},\cdots ,p^{N}\right\}. \end{array}$$

where conv{·} represents the convex hull.

Remark 2 Performance 1 describes the features of the functions $u_{ij}\left(\cdot \right)$ and $u_{i*}\left(\cdot \right)$. The action of UGV j on i and the action of UGV i on j are equal in magnitude and opposite in direction. Performance 2 shows that the swarm size is bounded, which also reflects the aggregation and formation characteristics of the swarm. Performance 3 depicts the cooperative hunting feature and swarm tracking capability, wherein all UGVs could enclose and surround the target.

## 3 Dynamic model with uncertainty and cyber interference

The dynamic model of UGV swarm system with uncertainty and cyber interference can be expressed as

(13) $$\begin{array}{ccc} & & M_{i}\left(p^{i}\left(t\right),\varrho_{i}\left(t\right),t\right){\ddot{p}}^{i}\left(t\right)+C_{i}\left(p^{i}\left(t\right),{\dot{p}}^{i}\left(t\right),\varrho_{i}\left(t\right),t\right)\\ & & \;\;\times \;{\dot{p}}^{i}\left(t\right)+G_{i}\left(p^{i}\left(t\right),\varrho_{i}\left(t\right),t\right)+D_{ai}\left(p^{i}\left(t\right),{\dot{p}}^{i}\left(t\right),t\right)\nu_{ai}\\ & & \;\;=\phi_{i}\left(t\right)\tau_{i}\left(t\right), \end{array}$$

wherein $\varrho_{i}\left(t\right)\in \Gamma_{i}\subset R^{p}$ denotes the uncertain parameters, $M_{i}\left(p^{i},\varrho_{i},t\right)$ denotes the inertial matrix, $C_{i}\left(p^{i},{\dot{p}}^{i},\varrho_{i},t\right){\dot{p}}^{i}$ denotes the Coriolis/centrifugal terms, $G_{i}\left(p^{i},\varrho_{i},t\right)$ denotes the gravitational terms; the $D_{ai}\left(p^{i}\left(t\right),{\dot{p}}^{i}\left(t\right),t\right)$ is the input matrix of cyber interference, $\nu_{ai}\in \Sigma_{i}\subset R^{l}$ is the unknown cyber interference input, $\tau_{i}\left(t\right)$ is the control input, $\phi_{i}\left(t\right)$ is the uncertain factor due to cyber interference (such as Denial of Service, spoofing and/or the mixed threat interferences). Matrices/vectors $M_{i}\left(p^{i},\varrho_{i},t\right)$, $C_{i}\left(p^{i},{\dot{p}}^{i},\varrho_{i},t\right){\dot{p}}^{i}$, $G_{i}\left(p^{i},\varrho_{i},t\right)$ and $D_{ai}\left(p^{i},{\dot{p}}^{i},t\right)$ are of appropriate dimensions. Functions $M_{i}\left(\cdot \right)$, $C_{i}\left(\cdot \right)$, $G_{i}\left(\cdot \right)$ and $D_{ai}\left(\cdot \right)$ are all continuous.

Inspired by the Udwadia-Kalaba constraint following modeling method, the integrated kinematic model (1) can be viewed as a series of constraints [24], [25], [26]. Suppose the constraints imposed on the independent ith UGV can be rewritten as

(14) $$\begin{array}{c} \sum_{j=1}^{n}A_{lj}^{i}\left(p^{i},t\right){\dot{p}}_{j}^{i}=c_{l}^{i}\left(p^{i},t\right),\;l=1,2,\cdots ,m, \end{array}$$

where $p^{i}={\left[p_{1}^{i},p_{2}^{i},\cdots ,p_{n}^{i}\right]}^{T}$, m≤n, $A_{lj}^{i}\left(\cdot \right)$ and $c_{l}^{i}\left(\cdot \right)$ are both $C^{1}$.

They can also be reorganized with the matrix form

(15) $$\begin{array}{c} A_{i}\left(p^{i},t\right){\dot{p}}^{i}=c_{i}\left(p^{i},t\right), \end{array}$$

where $A_{i}={\left[A_{lj}^{i}\right]}^{m\times n}$, $c_{i}={\left[c_{1}^{i},c_{2}^{i},\cdots ,c_{m}^{i}\right]}^{T}$.

There are two perspectives to interpret these constraints. From one perspective, they are regarded as passively applied. That is, the corresponding constraint forces may be supplied by the external environment (or the system structure), which can enable the system to comply with the constraint condition. From another perspective, they are viewed as active. That is, the required forces are provided by the control input of the system to meet the constraints.

In contrast with the traditional integrating methods, here we perform differential operations on the constraints with respect to t:

(16) $$\begin{array}{ccc} & & \sum_{j=1}^{n}\frac{d}{dt}\left(A_{lj}^{i}\left(p^{i},t\right)\right){\dot{p}}_{j}^{i}+\sum_{j=1}^{n}A_{lj}^{i}\left(p^{i},t\right){\ddot{p}}_{j}^{i}\\ & & \;\;\;\;=\frac{d}{dt}c_{l}^{i}\left(p^{i},t\right),\;l=1,2,\cdots ,m. \end{array}$$

The second-order constraints can be expressed as

(17) $$\begin{array}{ccc} \sum_{j=1}^{n}A_{lj}^{i}\left(p^{i},t\right){\ddot{p}}_{j}^{i} & = & -\sum_{j=1}^{n}\frac{d}{dt}\left(A_{lj}^{i}\left(p^{i},t\right)\right){\dot{p}}_{j}^{i}+\frac{d}{dt}c_{l}^{i}\left(p^{i},t\right)\\ & = & :b_{l}^{i}\left(p^{i},{\dot{p}}^{i},t\right),\;l=1,2,\cdots ,m. \end{array}$$

Rewriting Eq. 17 into the matrix form yields

(18) $$\begin{array}{c} A_{i}\left(p^{i},t\right){\ddot{p}}^{i}=b_{i}\left(p^{i},{\dot{p}}^{i},t\right), \end{array}$$

where $b_{i}={\left[b_{1}^{i},b_{2}^{i},\cdots ,b_{m}^{i}\right]}^{T}$.

Remark 3 The system model (13) is unsettled, wherein the control input $\tau_{i}$ remains to be determined. Eqs. 14-18 can be regarded as the procedure of constraint processing. Notice that this treatment method makes the constraints change fundamentally. Hence, in order to drive the system to present the ideal kinematic characteristics, we should take this issue into account. That is, supplementary item shall be added in the control design to make up for this. The details will be stated in the next section.

## 4 Cooperative control design

Taking both uncertainty and cyber interference into account, we propose a class of cooperative controls in this section. The resulting control should guarantee the UGV swarm system to achieve the kinematic performances. Next, we will decompose the system uncertainty and cyber interference input matrix respectively to serve for the control design.

### 4.1 Uncertainty decomposition

Assumption 1 The inertial matrix $M_{i}\left(p^{i},\varrho_{i},t\right)$ is positive definite for each $\left(p^{i},t\right)\in R^{k}\times R$, $\varrho_{i}\in \Gamma_{i}$.

In most applications, this assumption is valid or even can be regarded as a common sense to some degree. Here we give it in the form of assumption for more accurate explanation. On this basis, let ${\bar{M}}_{i}$ represents the nominal portions with ${\bar{M}}_{i}>0$, then the following decompositions and equations are valid.

(19) $$H_{i}\left(p^{i},t\right):={\bar{M}}_{i}^{-1}\left(p^{i},t\right),$$

(20) $$▵H_{i}\left(p^{i},\varrho_{i},t\right):=M_{i}^{-1}\left(p^{i},\varrho_{i},t\right)-{\bar{M}}_{i}^{-1}\left(p^{i},t\right),$$

(21) $$E_{i}\left(p^{i},\varrho_{i},t\right):={\bar{M}}_{i}\left(p^{i},t\right)M_{i}^{-1}\left(p^{i},\varrho_{i},t\right)-I,$$

(22) $$▵H_{i}\left(p^{i},\varrho_{i},t\right)=H_{i}\left(p^{i},t\right)E_{i}\left(p^{i},\varrho_{i},t\right).$$

Assumption 2 The matrix $A_{i}\left(p^{i},t\right)$ is of full rank for each $\left(p^{i},t\right)\in R^{k}\times R$, so the matrix $A_{i}\left(p^{i},t\right)A_{i}^{T}\left(p^{i},t\right)$ is invertible.

Based on Assumption 2, for the given positive definite matrix $K_{i}\in R^{m\times k}$, we define

(23) $$\begin{array}{ccc} \Phi_{i}\left(p^{i},\varrho_{i},t\right) & = & K_{i}A_{i}\left(p^{i},t\right)H_{i}\left(p^{i},t\right)E_{i}\left(p^{i},\varrho_{i},t\right){\bar{M}}_{i}\left(p^{i},t\right)\\ & & \times A_{i}^{T}\left(p^{i},t\right){\left(A_{i}\left(p^{i},t\right)A_{i}^{T}\left(p^{i},t\right)\right)}^{-1}K_{i}^{-1}. \end{array}$$

Assumption 3 For all $\left(p^{i},t\right)\in R^{k}\times R$, there exists a scalar $\delta_{E_{i}}$ such that

(24) $$\begin{array}{c} \min_{\varrho_{i}\in \Gamma_{i}}\lambda_{min}\left[\Phi_{i}\left(p^{i},\varrho_{i},t\right)+\Phi_{i}^{T}\left(p^{i},\varrho_{i},t\right)\right]\geq 2\delta_{E_{i}}>-2. \end{array}$$

Remark 4 The bound of the uncertainty is unknown, so the value of constant $\delta_{E_{i}}$ is unknown. If there is no uncertainty in the system, then we have $\delta_{E_{i}}=0$. Based on the continuity theory, this assumption imposes the influence of uncertainty within a certain threshold, and this threshold is unidirectional.

### 4.2 Cyber interference input matrix decomposition

With Assumption 2, we can derive the $\left(A_{i}H_{i}\right){\left(A_{i}H_{i}\right)}^{T}$ is invertible, then we have the following decompositions:

$$\begin{array}{c} D_{ai}={\hat{D}}_{ai}+{\tilde{D}}_{ai}. \end{array}$$

where

$$\begin{array}{ccc} {\hat{D}}_{ai} & = & {\left(A_{i}H_{i}\right)}^{T}{\left[\left(A_{i}H_{i}\right){\left(A_{i}H_{i}\right)}^{T}\right]}^{-1}A_{i}H_{i}D_{ai},\\ {\tilde{D}}_{ai} & = & D_{ai}-{\left(A_{i}H_{i}\right)}^{T}{\left[\left(A_{i}H_{i}\right){\left(A_{i}H_{i}\right)}^{T}\right]}^{-1}A_{i}H_{i}D_{ai}. \end{array}$$

Lemma 1 *For any uncertain vector* ν*, it is within the zero space of the matrix* $A_{i}H_{i}{\tilde{D}}_{ai}$*. That is,*

(25) $$\begin{array}{c} A_{i}H_{i}{\tilde{D}}_{ai}\nu \equiv 0. \end{array}$$

Proof $$\begin{array}{ccc} A_{i}H_{i}{\tilde{D}}_{ai}\nu & = & A_{i}H_{i}\left(D_{ai}-{\hat{D}}_{ai}\right)\nu \\ & = & A_{i}H_{i}(D_{ai}\nu -{\left(A_{i}H_{i}\right)}^{T}{\left[\left(A_{i}H_{i}\right){\left(A_{i}H_{i}\right)}^{T}\right]}^{-1}\\ & & \times A_{i}H_{i}D_{ai})\nu \\ & = & A_{i}H_{i}D_{ai}\nu -A_{i}H_{i}{\left(A_{i}H_{i}\right)}^{T}\\ & & \times {\left[\left(A_{i}H_{i}\right){\left(A_{i}H_{i}\right)}^{T}\right]}^{-1}A_{i}H_{i}D_{ai}\nu \\ & = & \left(A_{i}H_{i}D_{ai}-A_{i}H_{i}D_{ai}\right)\nu =0. \end{array}$$

Q.E.D. □

Remark 5 Based on the above decompositions, the uncertain cyber interference input $\nu_{ai}$ through the matrix ${\tilde{D}}_{ai}$ will not influence the system performance, which can be utilized in the next control design.

### 4.3 Adaptive robust control design

For the UGV swarm system with uncertainty and cyber interference input, we propose a cooperative adaptive robust control method.

Assumption 4 There exists a positive lower bound (known or unknown) ${\bar{\phi }}_{i}$ of the uncertain factor $\phi_{i}\left(t\right)$, such that $0<{\bar{\phi }}_{i}\leq \phi_{i}\left(t\right)\leq 1$ for all t∈R.

Assumption 5 Let $F_{i}\left(p^{i},{\dot{p}}^{i},\varrho_{i},t\right)=-C_{i}\left(p^{i},{\dot{p}}^{i},\varrho_{i},t\right){\dot{p}}^{i}-G_{i}\left(p^{i},\varrho_{i},t\right)$ and then we make the following assumptions.

(i) There exists an unknown constant vector $\gamma_{i}\in {\left(0,\infty \right)}^{\rho_{i}}$ and a known function $\Theta_{i}\left(\cdot \right)$: ${\left(0,\infty \right)}^{\rho_{i}}\times R^{n}\times R^{n}\times R\to R_{+}^{l}$, such that

(26) $$\begin{array}{ccc} & & {\left(1+\delta_{E_{i}}\right)}^{-1}\max_{\varrho_{i}\in \Gamma_{i},\nu_{ai}\in \Sigma_{i}}∥K_{i}A_{i}\left(p^{i},t\right)[M_{i}^{-1}\left(p^{i},\varrho_{i},t\right)F_{i}\left(p^{i},{\dot{p}}^{i},\varrho_{i},t\right)\\ & & \;\;-H_{i}\left(p^{i},t\right){\hat{D}}_{ai}\left(p^{i},{\dot{p}}^{i},t\right)\nu_{ai}-\Delta H_{i}\left(p^{i},t\right)\\ & & \;\;\times D_{ai}\left(p^{i},{\dot{p}}^{i},t\right)\nu_{ai}]-K_{i}b_{i}∥\leq \Theta_{i}\left(\gamma_{i},p^{i},{\dot{p}}^{i},t\right), \end{array}$$

for all $\left(p^{i},{\dot{p}}^{i},\varrho_{i},t\right)\in R^{n}\times R^{n}\times \Gamma_{i}\times R$.

(ii) The function $\Theta_{i}\left(\cdot \right)$ is continuous, concave and nondecreasing with respect to each element of $\gamma_{i}$. For any $\gamma_{i1}\in {\left(0,\infty \right)}^{\rho_{i}}$ and $\gamma_{i2}\in {\left(0,\infty \right)}^{\rho_{i}}$,

(27) $$\begin{array}{c} \Theta_{i}\left(\gamma_{i1},p^{i},{\dot{p}}^{i},t\right)-\Theta_{i}\left(\gamma_{i2},p^{i},{\dot{p}}^{i},t\right)\leq \frac{\partial \Theta_{i}}{\partial \gamma_{i}}\left(\gamma_{i2},p^{i},{\dot{p}}^{i},t\right)\left(\gamma_{i1}-\gamma_{i2}\right). \end{array}$$

Remark 6 The function $\Theta_{i}\left(\cdot \right)$ describes the structure of the uncertainty bound. The assumption parameterizes the worst-case effects associated with uncertainty.

Define constraint-following error $\epsilon_{i}=A_{i}\left(p^{i},t\right){\dot{p}}^{i}-c_{i}\left(p^{i},t\right)$. With the above assumptions, we define the following two portions

(28) $$\begin{array}{ccc} s_{i1}\left(p^{i},{\dot{p}}^{i},t\right) & = & -\kappa_{i}{\bar{M}}_{i}\left(p^{i},t\right)A_{i}^{T}\left(p^{i},t\right){\left[A_{i}\left(p^{i},t\right)A_{i}^{T}\left(p^{i},t\right)\right]}^{-1}\\ & & \times K_{i}^{-1}\epsilon_{i}\left(p^{i},{\dot{p}}^{i},t\right), \end{array}$$

(29) $$\begin{array}{ccc} s_{i2}\left({\hat{\gamma }}_{i},p^{i},{\dot{p}}^{i},t\right) & = & -{\bar{M}}_{i}\left(p^{i},t\right)A_{i}^{T}\left(p^{i},t\right){\left(A_{i}\left(p^{i},t\right)A_{i}^{T}\left(p^{i},t\right)\right)}^{-1}\\ & & \times K_{i}^{-1}\epsilon_{i}\left(p^{i},{\dot{p}}^{i},t\right)\Theta_{i}^{2}\left({\hat{\gamma }}_{i},p^{i},{\dot{p}}^{i},t\right), \end{array}$$

(30) $$\begin{array}{c} \dot{\hat{\gamma_{i}}}=Y_{i1}\frac{\partial \Theta_{i}^{T}}{\partial \gamma_{i}}\left({\hat{\gamma }}_{i},p^{i},{\dot{p}}^{i},t\right)∥\epsilon_{i}∥-Y_{i2}{\hat{\gamma }}_{i}, \end{array}$$

where $\kappa_{i}$ denotes positive scalar design parameter; the $Y_{i1}\in R^{\rho_{i}\times \rho_{i}}$ and $Y_{i2}\in R^{\rho_{i}\times \rho_{i}}$ are all positive definite; ${\hat{\gamma }}_{i}$ denotes the estimated value of $\gamma_{i}$, with the initial condition that $\gamma_{i}\left(t_{0}\right)>0$.

As a result, the cooperative adaptive robust control can be designed as

(31) $$\begin{array}{cc} \tau_{i}\left(t\right)= & s_{i1}\left(p^{i}\left(t\right),{\dot{p}}^{i}\left(t\right),t\right)+s_{i2}\left({\hat{\gamma }}_{i},p^{i}\left(t\right),{\dot{p}}^{i}\left(t\right),t\right). \end{array}$$

where the nominal feedback control term $s_{i1}$ can direct the swarm system towards the desirable constraints and stabilize the nominal system, and the adaptive robust control term $s_{i2}$ is to counteract the interference of uncertainty and cyber interference input, which can be tuned in real time by the value of the adaptive parameter $\hat{\gamma_{i}}$.

Theorem 1 *Let* $\alpha_{i}:=\left[\epsilon_{i}^{T},{\left({\hat{\gamma }}_{i}-\gamma_{i}\right)}^{T}\right]{}^{T}$*,* $\alpha :=[\alpha_{1}^{T},\alpha_{2}^{T},$ $\cdots ,\alpha_{n}^{T}]{}^{T}$*. Subject to the previous assumptions, the cooperative control* $\tau_{i}\left(t\right)$ *renders the system error* α(t) *uniformly bounded (UB) and uniformly ultimately bounded (UUB).*

Proof The Lyapunov function candidate can be chosen as

(32) $$\begin{array}{c} V=\sum_{i=1}^{n}V_{i}, \end{array}$$

where $V_{i}=\frac{1}{2}\epsilon_{i}^{T}K_{i}\epsilon_{i}+\frac{1}{2}\left(1+\delta_{E_{i}}\right){\left({\hat{\gamma }}_{i}-\gamma_{i}\right)}^{T}Y_{i1}^{-1}\left({\hat{\gamma }}_{i}-\gamma_{i}\right)$.

For better differentiation, we define

(33) $$V_{i1}=\frac{1}{2}\epsilon_{i}^{T}K_{i}\epsilon_{i},$$

(34) $$V_{i2}=\frac{1}{2}\left(1+\delta_{E_{i}}\right){\left({\hat{\gamma }}_{i}-\gamma_{i}\right)}^{T}\eta_{i1}^{-1}\left({\hat{\gamma }}_{i}-\gamma_{i}\right).$$

Taking the derivative of $V_{i1}$ yields

(35) $$\begin{array}{ccc} {\dot{V}}_{i1} & = & \epsilon_{i}^{T}K_{i}{\dot{\epsilon }}_{i}\\ & = & \epsilon_{i}^{T}K_{i}\left(A_{i}{\ddot{p}}^{i}-b_{i}\right)\\ & = & \epsilon_{i}^{T}K_{i}\left\{A_{i}\left[M_{i}^{-1}\left(-C_{i}{\dot{p}}^{i}-G_{i}-D_{ai}\nu_{ai}+\phi_{i}\tau_{i}\right)\right]-b_{i}\right\}\\ & = & \epsilon_{i}^{T}K_{i}\left\{A_{i}[M_{i}^{-1}\left(-C_{i}{\dot{p}}^{i}-G_{i}-D_{ai}\nu_{ai}+\phi_{i}\left(s_{i1}+s_{i2}\right)\right)]-b_{i}\right\}. \end{array}$$

Since $M_{i}^{-1}:=H_{i}+\Delta H_{i}$ and $D_{ai}:={\hat{D}}_{ai}+{\tilde{D}}_{ai}$, the reorganized terms can be written as

(36) $$\begin{array}{ccc} & & A_{i}\left[M_{i}^{-1}\left(-C_{i}{\dot{p}}^{i}-G_{i}-D_{ai}\nu_{ai}+\phi_{i}\left(s_{i1}+s_{i2}\right)\right)\right]-b_{i}\\ & & \;=A_{i}M_{i}^{-1}F_{i}-A_{i}M_{i}^{-1}D_{ai}\nu_{ai}+A_{i}M_{i}^{-1}\phi_{i}\left(s_{i1}+s_{i2}\right)-b_{i}\\ & & \;=A_{i}M_{i}^{-1}F_{i}-A_{i}\left(H_{i}+\Delta H_{i}\right)\left({\hat{D}}_{ai}+{\tilde{D}}_{ai}\right)\nu_{ai}+A_{i}(H_{i}+\\ & & \;\Delta H_{i})\phi_{i}\left(s_{i1}+s_{i2}\right)-b_{i}. \end{array}$$

By Eq. 25, $A_{i}H_{i}\left({\hat{D}}_{ai}+{\tilde{D}}_{ai}\right)\nu_{ai}=A_{i}H_{i}{\hat{D}}_{ai}\nu_{ai}$, then we have

(37) $$\begin{array}{ccc} & & A_{i}M_{i}^{-1}F_{i}-A_{i}\left(H_{i}+\Delta H_{i}\right)\left({\hat{D}}_{ai}+{\tilde{D}}_{ai}\right)\nu_{ai}-b_{i}\\ & & \;=A_{i}M_{i}^{-1}F_{i}-A_{i}H_{i}{\hat{D}}_{ai}\nu_{ai}-A_{i}\Delta H_{i}D_{ai}\nu_{ai}-b_{i}. \end{array}$$

Based on Eq. 26,

(38) $$\begin{array}{ccc} & & \epsilon_{i}^{T}K_{i}\left\{A_{i}\left[M_{i}^{-1}\left(-C_{i}{\dot{p}}^{i}-G_{i}-D_{ai}\nu_{ai}\right)\right]-b_{i}\right\}\\ & & \;=\epsilon_{i}^{T}K_{i}\left\{A_{i}\left[M_{i}^{-1}F_{i}-\left(H_{i}+\Delta H_{i}\right)D_{ai}\nu_{ai}\right]-b_{i}\right\}\\ & & \;=\epsilon_{i}^{T}K_{i}\left\{A_{i}\left[M_{i}^{-1}F_{i}-\left(H_{i}{\hat{D}}_{ai}+\Delta H_{i}D_{ai}\right)\nu_{ai}\right]-b_{i}\right\}\\ & & \;\leq ∥\epsilon_{i}∥∥K_{i}A_{i}\left[M_{i}^{-1}F_{i}-\left(H_{i}{\hat{D}}_{ai}+\Delta H_{i}D_{ai}\right)\nu_{ai}\right]-K_{i}b_{i}∥\\ & & \;\leq ∥\epsilon_{i}∥\left(1+\delta_{E_{i}}\right)\Theta_{i}\left(\gamma_{i},p^{i},{\dot{p}}^{i},t\right). \end{array}$$

 □

The rest terms relevant to $s_{i1}$ can be converted to

(39) $$\begin{array}{ccc} & & \epsilon_{i}^{T}K_{i}A_{i}\left(H_{i}+\Delta H_{i}\right)\phi_{i}s_{i1}\\ & & \;=\epsilon_{i}^{T}K_{i}A_{i}H_{i}\phi_{i}s_{i1}+\epsilon_{i}^{T}K_{i}\Delta H_{i}\phi_{i}s_{i1}\\ & & \;=\epsilon_{i}^{T}K_{i}A_{i}H_{i}\phi_{i}\left[-\kappa_{i}{\bar{M}}_{i}A_{i}^{T}{\left(A_{i}A_{i}^{T}\right)}^{-1}K_{i}^{-1}\epsilon_{i}\right]\\ & & \;+\;\epsilon_{i}^{T}K_{i}A_{i}\Delta H_{i}\phi_{i}\left[-\kappa_{i}{\bar{M}}_{i}A_{i}^{T}{\left(A_{i}A_{i}^{T}\right)}^{-1}K_{i}^{-1}\epsilon_{i}\right]. \end{array}$$

Recalling ${\bar{M}}_{i}^{-1}=H_{i}$ and performing matrix operations,

(40) $$\begin{array}{ccc} & & \epsilon_{i}^{T}K_{i}A_{i}H_{i}\phi_{i}\left[-\kappa_{i}{\bar{M}}_{i}A_{i}^{T}{\left(A_{i}A_{i}^{T}\right)}^{-1}K_{i}^{-1}\epsilon_{i}\right]\\ & & \;=-\kappa_{i}\phi_{i}\epsilon_{i}^{T}\left[K_{i}A_{i}H_{i}{\bar{M}}_{i}A_{i}^{T}{\left(A_{i}A_{i}^{T}\right)}^{-1}K_{i}^{-1}\right]\epsilon_{i}\\ & & \;\leq -\kappa_{i}{\bar{\phi }}_{i}{∥\epsilon_{i}∥}^{2}. \end{array}$$

Based on Eqs. 23 and 24, the rest terms can be rewritten as

(41) $$\begin{array}{ccc} & & \epsilon_{i}^{T}K_{i}A_{i}\Delta H_{i}\phi_{i}\left[-\kappa_{i}{\bar{M}}_{i}A_{i}^{T}{\left(A_{i}A_{i}^{T}\right)}^{-1}K_{i}^{-1}\epsilon_{i}\right]\\ & & \;=-\kappa_{i}\phi_{i}\epsilon_{i}^{T}\left[K_{i}A_{i}H_{i}E_{i}{\bar{M}}_{i}A_{i}^{T}{\left(A_{i}A_{i}^{T}\right)}^{-1}K_{i}^{-1}\right]\epsilon_{i}\\ & & \;\leq -\kappa_{i}{\bar{\phi }}_{i}\epsilon_{i}^{T}\frac{1}{2}\left[\Phi_{i}+\Phi_{i}^{T}\right]\epsilon_{i}\\ & & \;\leq -\kappa_{i}{\bar{\phi }}_{i}\frac{1}{2}\lambda_{min}\left[\Phi_{i}+\Phi_{i}^{T}\right]{∥\epsilon_{i}∥}^{2}\\ & & \;\leq -\kappa_{i}{\bar{\phi }}_{i}\delta_{E_{i}}{∥\epsilon_{i}∥}^{2}. \end{array}$$

Combining Eqs. 40 and 41, we have

(42) $$\begin{array}{c} \epsilon_{i}^{T}K_{i}A_{i}\left(H_{i}+\Delta H_{i}\right)\phi_{i}s_{i1}\leq -\kappa_{i}{\bar{\phi }}_{i}\left(1+\delta_{E_{i}}\right){∥\epsilon_{i}∥}^{2}. \end{array}$$

The rest terms relating to $s_{i2}$ can be rewritten as

(43) $$\begin{array}{ccc} & & \epsilon_{i}^{T}K_{i}A_{i}\left(H_{i}+\Delta H_{i}\right)\phi_{i}s_{i2}\\ & & =\epsilon_{i}^{T}K_{i}A_{i}H_{i}\phi_{i}\left[-{\bar{M}}_{i}A_{i}^{T}{\left(A_{i}A_{i}^{T}\right)}^{-1}K_{i}^{-1}\epsilon_{i}\Theta_{i}^{2}\left({\hat{\gamma }}_{i}\right)\right]\\ & & \;+\;\epsilon_{i}^{T}K_{i}A_{i}H_{i}E_{i}\phi_{i}\left[-{\bar{M}}_{i}A_{i}^{T}{\left(A_{i}A_{i}^{T}\right)}^{-1}K_{i}^{-1}\epsilon_{i}\Theta_{i}^{2}\left({\hat{\gamma }}_{i}\right)\right]. \end{array}$$

Based on the matrix operations,

(44) $$\begin{array}{ccc} & & \epsilon_{i}^{T}K_{i}A_{i}H_{i}\phi_{i}\left[-{\bar{M}}_{i}A_{i}^{T}{\left(A_{i}A_{i}^{T}\right)}^{-1}K_{i}^{-1}\epsilon_{i}\Theta_{i}^{2}\left({\hat{\gamma }}_{i}\right)\right]\\ & & \;=-\phi_{i}\epsilon_{i}^{T}\left[K_{i}A_{i}H_{i}{\bar{M}}_{i}A_{i}^{T}{\left(A_{i}A_{i}^{T}\right)}^{-1}K_{i}^{-1}\right]\epsilon_{i}\Theta_{i}^{2}\left({\hat{\gamma }}_{i}\right)\\ & & \;\leq -{\bar{\phi }}_{i}{∥\epsilon_{i}∥}^{2}\Theta_{i}^{2}\left({\hat{\gamma }}_{i}\right). \end{array}$$

By a similar algebra as for $s_{i1}$, we can show that

(45) $$\begin{array}{ccc} & & \epsilon_{i}^{T}K_{i}A_{i}H_{i}E_{i}\phi_{i}\left[-{\bar{M}}_{i}A_{i}^{T}{\left(A_{i}A_{i}^{T}\right)}^{-1}K_{i}^{-1}\epsilon_{i}\Theta_{i}^{2}\left({\hat{\gamma }}_{i}\right)\right]\\ & & \;\leq -\phi_{i}\delta_{E_{i}}{∥\epsilon_{i}∥}^{2}\Theta_{i}^{2}\left({\hat{\gamma }}_{i}\right)\leq -{\bar{\phi }}_{i}\delta_{E_{i}}{∥\epsilon_{i}∥}^{2}\Theta_{i}^{2}\left({\hat{\gamma }}_{i}\right). \end{array}$$

Combining (44) and (45),

(46) $$\begin{array}{ccc} & & \epsilon_{i}^{T}K_{i}A_{i}\left(H_{i}+\Delta H_{i}\right)\phi_{i}s_{i2}\\ & & \;\leq -{\bar{\phi }}_{i}\left(1+\delta_{E_{i}}\right){∥\epsilon_{i}∥}^{2}\Theta_{i}^{2}\left({\hat{\gamma }}_{i}\right). \end{array}$$

Summarizing the above results, the evaluation of ${\dot{V}}_{i1}$ can be expressed as

(47) $$\begin{array}{ccc} {\dot{V}}_{i1} & = & \epsilon_{i}^{T}K_{i}{\dot{\epsilon }}_{i}\\ & \leq & ∥\epsilon_{i}∥\left(1+\delta_{E_{i}}\right)\Theta_{i}\left(\gamma_{i}\right)-\kappa_{i}{\bar{\phi }}_{i}\left(1+\delta_{E_{i}}\right){∥\epsilon_{i}∥}^{2}\\ & & -\;{\bar{\phi }}_{i}\left(1+\delta_{E_{i}}\right){∥\epsilon_{i}∥}^{2}\Theta_{i}^{2}\left({\hat{\gamma }}_{i}\right). \end{array}$$

Taking the derivative of $V_{i2}$ yields

(48) $$\begin{array}{ccc} {\dot{V}}_{i2} & = & \left(1+\delta_{E_{i}}\right){\left({\hat{\gamma }}_{i}-\gamma_{i}\right)}^{T}Y_{i1}^{-1}\dot{\hat{\gamma_{i}}}\\ & = & \left(1+\delta_{E_{i}}\right){\left({\hat{\gamma }}_{i}-\gamma_{i}\right)}^{T}\frac{\partial \Theta_{i}^{T}}{\partial \gamma_{i}}\left({\hat{\gamma }}_{i}\right)∥\epsilon_{i}∥\\ & & -\;\left(1+\delta_{E_{i}}\right){\left({\hat{\gamma }}_{i}-\gamma_{i}\right)}^{T}Y_{i1}^{-1}Y_{i2}{\hat{\gamma }}_{i}. \end{array}$$

Therefore,

(49) $$\begin{array}{ccc} {\dot{V}}_{i} & \leq & ∥\epsilon_{i}∥\left(1+\delta_{E_{i}}\right)\Theta_{i}\left(\gamma_{i}\right)-\kappa_{i}{\bar{\phi }}_{i}\left(1+\delta_{E_{i}}\right){∥\epsilon_{i}∥}^{2}\\ & & -{\bar{\phi }}_{i}\left(1+\delta_{E_{i}}\right){∥\epsilon_{i}∥}^{2}\Theta_{i}^{2}\left({\hat{\gamma }}_{i}\right)+\left(1+\delta_{E_{i}}\right){\left({\hat{\gamma }}_{i}-\gamma_{i}\right)}^{T}\\ & & \times \frac{\partial \Theta_{i}^{T}}{\partial \gamma_{i}}\left({\hat{\gamma }}_{i}\right)∥\epsilon_{i}∥-\left(1+\delta_{E_{i}}\right){\left({\hat{\gamma }}_{i}-\gamma_{i}\right)}^{T}Y_{i1}^{-1}Y_{i2}{\hat{\gamma }}_{i}\\ & = & ∥\epsilon_{i}∥\left(1+\delta_{E_{i}}\right)\Theta_{i}\left(\gamma_{i}\right)-∥\epsilon_{i}∥\left(1+\delta_{E_{i}}\right)\Theta_{i}\left({\hat{\gamma }}_{i}\right)\\ & & +∥\epsilon_{i}∥\left(1+\delta_{E_{i}}\right)\Theta_{i}\left({\hat{\gamma }}_{i}\right)-\kappa_{i}{\bar{\phi }}_{i}\left(1+\delta_{E_{i}}\right){∥\epsilon_{i}∥}^{2}\\ & & -{\bar{\phi }}_{i}\left(1+\delta_{E_{i}}\right){∥\epsilon_{i}∥}^{2}\Theta_{i}^{2}\left({\hat{\gamma }}_{i}\right)+\left(1+\delta_{E_{i}}\right){\left({\hat{\gamma }}_{i}-\gamma_{i}\right)}^{T}\\ & & \times \frac{\partial \Theta_{i}^{T}}{\partial \gamma_{i}}\left({\hat{\gamma }}_{i}\right)∥\epsilon_{i}∥-\left(1+\delta_{E_{i}}\right){\left({\hat{\gamma }}_{i}-\gamma_{i}\right)}^{T}Y_{i1}^{-1}Y_{i2}{\hat{\gamma }}_{i}\\ & \leq & ∥\epsilon_{i}∥\left(1+\delta_{E_{i}}\right)\Theta_{i}\left({\hat{\gamma }}_{i}\right)-{\bar{\phi }}_{i}\left(1+\delta_{E_{i}}\right){∥\epsilon_{i}∥}^{2}\Theta_{i}^{2}\left({\hat{\gamma }}_{i}\right)\\ & & -\kappa_{i}{\bar{\phi }}_{i}\left(1+\delta_{E_{i}}\right){∥\epsilon_{i}∥}^{2}-\left(1+\delta_{E_{i}}\right){\left({\hat{\gamma }}_{i}-\gamma_{i}\right)}^{T}Y_{i1}^{-1}Y_{i2}{\hat{\gamma }}_{i}\\ & \leq & -\kappa_{i}{\bar{\phi }}_{i}\left(1+\delta_{E_{i}}\right){∥\epsilon_{i}∥}^{2}+\frac{1+\delta_{E_{i}}}{4{\bar{\phi }}_{i}}-\left(1+\delta_{E_{i}}\right)\\ & & \times {\left({\hat{\gamma }}_{i}-\gamma_{i}\right)}^{T}Y_{i1}^{-1}Y_{i2}{\hat{\gamma }}_{i}. \end{array}$$

Since

(50) $$\begin{array}{cc} & -\left(1+\delta_{E_{i}}\right){\left({\hat{\gamma }}_{i}-\gamma_{i}\right)}^{T}Y_{i1}^{-1}Y_{i2}{\hat{\gamma }}_{i}\\ & \;=-\left(1+\delta_{E_{i}}\right){\left({\hat{\gamma }}_{i}-\gamma_{i}\right)}^{T}Y_{i1}^{-1}Y_{i2}\left({\hat{\gamma }}_{i}-\gamma_{i}+\gamma_{i}\right)\\ & \;=-\left(1+\delta_{E_{i}}\right){\left({\hat{\gamma }}_{i}-\gamma_{i}\right)}^{T}Y_{i1}^{-1}Y_{i2}\left({\hat{\gamma }}_{i}-\gamma_{i}\right)\\ & \;-\left(1+\delta_{E_{i}}\right){\left({\hat{\gamma }}_{i}-\gamma_{i}\right)}^{T}Y_{i1}^{-1}Y_{i2}\gamma_{i}\\ & \;\leq -\left(1+\delta_{E_{i}}\right)\lambda_{min}\left(Y_{i1}^{-1}Y_{i2}\right){∥{\hat{\gamma }}_{i}-\gamma_{i}∥}^{2}\\ & \;+\left(1+\delta_{E_{i}}\right)∥Y_{i1}^{-1}Y_{i2}\gamma_{i}∥∥{\hat{\gamma }}_{i}-\gamma_{i}∥. \end{array}$$

The overall evaluation can be expressed as

(51) $$\begin{array}{cc} {\dot{V}}_{i}\leq & -\kappa_{i}{\bar{\phi }}_{i}\left(1+\delta_{E_{i}}\right){∥\epsilon_{i}∥}^{2}+\frac{1+\delta_{E_{i}}}{4{\bar{\phi }}_{i}}\\ & -\left(1+\delta_{E_{i}}\right)\lambda_{min}\left(Y_{i1}^{-1}Y_{i2}\right){∥{\hat{\gamma }}_{i}-\gamma_{i}∥}^{2}\\ & +\left(1+\delta_{E_{i}}\right)∥Y_{i1}^{-1}Y_{i2}\gamma_{i}∥∥{\hat{\gamma }}_{i}-\gamma_{i}∥. \end{array}$$

We let ${\hat{z}}_{i1}:=\min\left\{\kappa_{i}{\bar{\phi }}_{i}\left(1+\delta_{E_{i}}\right),\left(1+\delta_{E_{i}}\right)\lambda_{\min}\left(Y_{i1}^{-1}Y_{i2}\right)\right\}$, ${\hat{z}}_{i2}:=$ $\left(1+\delta_{E_{i}}\right)∥Y_{i1}^{-1}Y_{i2}\gamma_{i}∥$, ${\hat{z}}_{i3}:=\left(1+\delta_{E_{i}}\right)/\left(4{\bar{\phi }}_{i}\right)$, then

(52) $$\begin{array}{c} {\dot{V}}_{i}\leq -{\hat{z}}_{i1}∥\alpha_{i}{∥}^{2}+{\hat{z}}_{i2}∥\alpha_{i}∥+{\hat{z}}_{i3}. \end{array}$$

Let ${\hat{z}}_{1}=\min_{i\in N}{\hat{z}}_{i1}$, ${\hat{z}}_{2}=\max_{i\in N}{\hat{z}}_{i2}$, ${\hat{z}}_{3}=\max_{i\in N}{\hat{z}}_{i3}$. As a result,

(53) $$\begin{array}{cc} \dot{V}= & \sum_{i=1}^{n}{\dot{V}}_{i}\leq -{\hat{z}}_{1}{∥\alpha ∥}^{2}+n{\hat{z}}_{2}∥\alpha ∥+n{\hat{z}}_{3}. \end{array}$$

We define

$$\begin{array}{c} {\hat{\rho }}_{i1}:=\frac{1}{2}\min_{i\in N}\left\{\lambda_{min}\left(K_{i}\right),\left(1+\delta_{E_{i}}\right)\lambda_{min}\left(Y_{i1}^{-1}\right)\right\},\\ {\hat{\rho }}_{i2}:=\frac{1}{2}\max_{i\in N}\left\{\lambda_{max}\left(K_{i}\right),\left(1+\delta_{E_{i}}\right)\lambda_{max}\left(Y_{i1}^{-1}\right)\right\}. \end{array}$$

By Rayleigh’s principle, we have

(54) $$\begin{array}{c} {\hat{\rho }}_{i1}{∥\alpha_{i}∥}^{2}\leq V_{i}\leq {\hat{\rho }}_{i2}{∥\alpha_{i}∥}^{2}. \end{array}$$

Let ${\hat{\rho }}_{1}=\min_{i\in N}\left\{{\hat{\rho }}_{i1}\right\}$, ${\hat{\rho }}_{2}=\max_{i\in N}\left\{{\hat{\rho }}_{i2}\right\}$, we have

(55) $$\begin{array}{c} {\hat{\rho }}_{1}{∥\alpha ∥}^{2}\leq V\leq {\hat{\rho }}_{2}{∥\alpha ∥}^{2}. \end{array}$$

The uniform boundedness can be guaranteed with

(56) $$d\left(r\right)=\left\{ \begin{array}{cc} \sqrt{\frac{{\hat{\rho }}_{2}}{{\hat{\rho }}_{1}}}R, & \;\;\;\;\;\text{if}\;r\leq R,\\ \sqrt{\frac{{\hat{\rho }}_{2}}{{\hat{\rho }}_{1}}}r, & \;\;\;\;\;\text{if}\;r>R. \end{array}\right.$$

where

(57) $$\begin{array}{c} R=\frac{n{\hat{z}}_{2}+\sqrt{n^{2}{\hat{z}}_{2}^{2}+4n{\hat{z}}_{1}{\hat{z}}_{3}}}{2{\hat{z}}_{1}}. \end{array}$$

The uniform ultimate boundedness can also be demonstrated with

(58) $$\begin{array}{c} \underset{‾}{d}=\sqrt{\frac{{\hat{\rho }}_{2}}{{\hat{\rho }}_{1}}}R, \end{array}$$

(59) $$T\left(\bar{d},r\right)=\left\{ \begin{array}{cc} 0, & \;\;\;\;\;\text{if}\;r\leq \bar{R},\\ \frac{{\hat{\rho }}_{2}r^{2}-{\hat{\rho }}_{1}{\bar{R}}^{2}}{{\hat{z}}_{1}{\bar{R}}^{2}-n{\hat{z}}_{2}\bar{R}-n{\hat{z}}_{3}}, & \;\;\;\;\;\text{if}\;r>\bar{R}. \end{array}\right.$$

and

(60) $$\begin{array}{c} \bar{R}=\sqrt{\frac{{\hat{\rho }}_{1}}{{\hat{\rho }}_{2}}}\bar{d}. \end{array}$$

Q.E.D.

Remark 7 The UB performance guarantees that the control error α can converge within a small region d(r). The UUB performance ensures that the error α can be sufficiently small. That is, after a finite time $T(\bar{d},r),$ the error can converge within a region $\left|\alpha \right|\leq \bar{d}.$ The uniform ultimate boundedness not only demonstrates that the cooperative control assures the system stability, but also indicates that the $∥\alpha_{i}∥$ finally enters a region smaller than the initial value under the proposed control.

## 5 Performance analysis

Definition 1 For all $t>t_{0}$, $\varrho_{i}\in \Gamma_{i}$ and $\nu_{ai}\in \Sigma_{i}$, if there exists a region D satisfying |α(t)|≤D, the controlled system is viewed robust to the interference factor $\phi_{i}$ and the cyber interference input $\nu_{ai}$.

Based on the characteristics of UB and UUB, we continue to explore the impacts on the robust performance of the swarm system.

### 5.1 No cyber interference input

When the swarm system is not influenced by the cyber interference, then the intact control input can be imposed on the system, i.e., $\phi_{i}\left(t\right)\equiv 1$, $\nu_{ai}\equiv 0$. In such scenarios, ${\hat{z}}_{i1}^{{}^{\prime }}:=\min\left\{\kappa_{i}\left(1+\delta_{E_{i}}\right),\left(1+\delta_{E_{i}}\right)\lambda_{\min}\left(Y_{i1}^{-1}Y_{i2}\right)\right\}$, ${\hat{z}}_{i2}^{{}^{\prime }}:=$ $\left(1+\delta_{E_{i}}\right)∥Y_{i1}^{-1}Y_{i2}\gamma_{i}∥$, ${\hat{z}}_{i3}^{{}^{\prime }}:=\left(1+\delta_{E_{i}}\right)/4$, which are corresponding to the new ${\hat{z}}_{1}^{{}^{\prime }}$, ${\hat{z}}_{2}^{{}^{\prime }}$, ${\hat{z}}_{3}^{{}^{\prime }}$, so the value of the R can be denoted as

(61) $$\begin{array}{c} R^{{}^{\prime }}=\frac{n{\hat{z}}_{2}^{{}^{\prime }}+\sqrt{{\left(n{\hat{z}}_{2}^{{}^{\prime }}\right)}^{2}+4n{\hat{z}}_{1}^{{}^{\prime }}{\hat{z}}_{3}^{{}^{\prime }}}}{2{\hat{z}}_{1}^{{}^{\prime }}}. \end{array}$$

For determined $Y_{i1}$, $Y_{i2}$ and $\delta_{E_{i}}$, the desirable value of $R^{{}^{\prime }}$ can be obtained by a suitable choice of $\kappa_{i}$.

### 5.2 Considering the cyber interference

If the swarm system suffers from cyber interference, the value of R can be acquired based on (57). In the case, $\phi_{i}\leq 1$, and $\gamma_{i}$ should account for the interference influence, which implies a greater ${\hat{z}}_{2}$. As a result, the value of R in (57) is greater than that of $R^{{}^{\prime }}$, which means the degradation of the constraint following performance compared with the case 5.1. Moreover, it is worth noting that R in (57) will always exist for any ${\bar{\phi }}_{i}>0$ and finite $\gamma_{i}$ (which relies on the bounds of $\varrho_{i}$ and interference input $\nu_{ai}$). This implies the robustness of the controlled swarm system when suffering from cyber interference.

## 6 An illustrative example

**Fig. 2:**
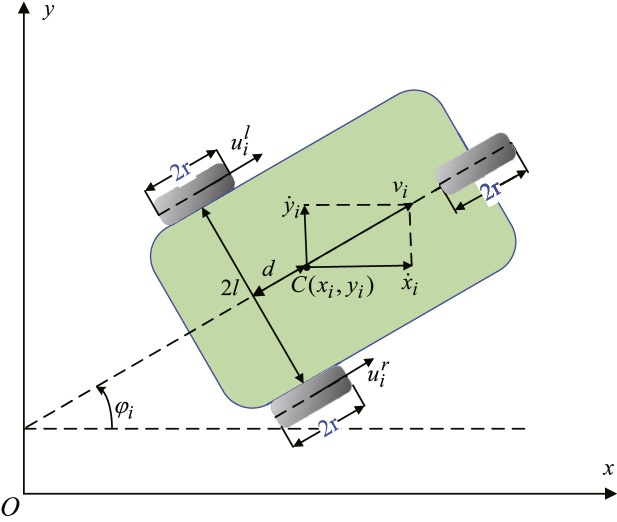
**Model of the**i**th vehicle**.

Consider a swarm system consisting of 6 homogeneous unmanned ground vehicles. Let $p^{i}={\left(x_{i},y_{i},\varphi_{i}\right)}^{T}$ denote the general coordinates of the ith UGV. The model of UGV i in the two-dimensional plane is shown in Fig. 2. There are two driving wheels mounted on the rear axis, and a front omni-directional wheel. The relevant parameters illustrations are shown in Table 1. Based on the Lagrangian methods, the dynamic model of UGV i can be formulated as

(62) $$\begin{array}{c} M_{i}{\ddot{p}}^{i}\left(t\right)+C_{i}{\dot{p}}^{i}\left(t\right)+G_{i}+D_{ai}\nu_{ai}\left(t\right)=\phi_{i}\left(t\right)\tau_{i}\left(t\right), \end{array}$$

where

$$\begin{array}{ccc} M_{i} & = & \left[\begin{array}{ccc} m_{i} & 0 & m_{i}ds_{i}\\ 0 & m_{i} & -m_{i}dc_{i}\\ m_{i}ds_{i} & -m_{i}dc_{i} & I_{i} \end{array}\right],\\ C_{i} & = & \left[\begin{array}{c} m_{i}d{\dot{\varphi }}_{i}^{2}c_{i}\\ m_{i}d{\dot{\varphi }}_{i}^{2}s_{i}\\ 0 \end{array}\right],G_{i}=\left[\begin{array}{c} s_{i}\lambda_{i}\\ -c_{i}\lambda_{i}\\ d\lambda_{i} \end{array}\right],\\ \tau_{i} & = & R_{i}u_{i}=\frac{1}{r}\left[\begin{array}{cc} c_{i} & c_{i}\\ s_{i} & s_{i}\\ l & -l \end{array}\right]\left[\begin{array}{c} u_{i}^{r}\\ u_{i}^{l} \end{array}\right],\\ \lambda_{i} & = & -m_{i}{\dot{\varphi }}_{i}\left(c_{i}{\dot{x}}_{i}+s_{i}{\dot{y}}_{i}\right), \end{array}$$

here $s_{i}=sin\varphi_{i}$, $c_{i}=cos\varphi_{i}$.

**Table 1:** **Descriptions of the system parameters**.

Notation/Unit	Description
$m_{i}/kg$	Mass
$I_{i}/kg.m^{2}$	Inertia moment
$\varphi_{i}/rad$	Orientation angle
2r/m	Diameter of the driving wheels
2l/m	Distance between two driving wheels
d/m	Distance between mass centre and rear axle
$u_{i}^{l}/N.m$	Driving torque of the left wheel (≤50N.m)
$u_{i}^{r}/N.m$	Driving torque of the right wheel (≤50N.m)
$x_{i}/m$	the position in the X-direction
$y_{i}/m$	the position in the Y-direction
${\dot{x}}_{i}/\left(m/s\right)$	the velocity in the X-direction
${\dot{y}}_{i}/\left(m/s\right)$	the velocity in the Y-direction
${\ddot{x}}_{i}/\left(m^{2}/s\right)$	the acceleration in the X-direction
${\ddot{y}}_{i}/\left(m^{2}/s\right)$	the acceleration in the Y-direction

Subject to the properties and ideal kinematic performances, the constraints can be designed as

(63) $$\begin{array}{c} \left[\begin{array}{c} {\dot{x}}_{i}\\ {\dot{y}}_{i} \end{array}\right]=-\sum_{j=1,j\neq i}^{n}u_{ij}-u_{i*}+\left[\begin{array}{c} {\dot{x}}_{*}\\ {\dot{y}}_{*} \end{array}\right], \end{array}$$

where

(64) $$\begin{array}{c} u_{ij}=\left[\begin{array}{c} x_{i}-x_{j}\\ y_{i}-y_{j} \end{array}\right]\left(a-\frac{b}{{\left(x_{i}-x_{j}\right)}^{2}+{\left(y_{i}-y_{j}\right)}^{2}+c}\right), \end{array}$$

(65) $$\begin{array}{c} u_{i*}=g\left[\begin{array}{c} x_{i}-x_{*}\\ y_{i}-y_{*} \end{array}\right]. \end{array}$$

here a, b, c and g are all positive scalar parameters.

The above constraint equations can be rewritten in the standard form of Eq. 15, so we have

(66) $$\begin{array}{cc} A_{i} & =\left[\begin{array}{ccc} 1 & 0 & 0\\ 0 & 1 & 0 \end{array}\right],\;{\dot{p}}^{i}={\left[\begin{array}{ccc} {\dot{x}}_{i} & {\dot{y}}_{i} & {\dot{\varphi }}_{i} \end{array}\right]}^{T},\\ c_{i} & =-\sum_{i=1}^{n}u_{ij}-u_{i*}+\left[\begin{array}{c} {\dot{x}}_{*}\\ {\dot{y}}_{*} \end{array}\right], \end{array}$$

(67) $$\begin{array}{cc} \epsilon_{i} & =A_{i}{\dot{p}}^{i}-c_{i}. \end{array}$$

Let ∥ϵ∥ denote the overall constraint following error, where $\epsilon ={\left[\epsilon_{1}^{T},\epsilon_{2}^{T},\epsilon_{3}^{T},\epsilon_{4}^{T},\epsilon_{5}^{T},\epsilon_{6}^{T}\right]}^{T}$. Let ∥u∥ denote the overall control cost, where $u={\left[u_{1}^{T},u_{2}^{T},u_{3}^{T},u_{4}^{T},u_{5}^{T},u_{6}^{T}\right]}^{T}$. For the specific time T, the average constraint following error $\bar{\epsilon }$ can be denoted as $\bar{\epsilon }=(\int_{0}^{T}∥\epsilon ∥)/T$, and the average control cost $\bar{u}$ can be denoted as $\bar{u}=(\int_{0}^{T}∥u∥)/T$.

We define $m_{i}={\bar{m}}_{i}+\Delta m_{i}$, $I_{i}={\bar{I}}_{i}+\Delta I_{i}$, here $\Delta m_{i}$ and $\Delta I_{i}$ denote the possibly time-varying uncertainty. For simulations, we set a=0.01, b=30, c=1, g=5, ${\bar{m}}_{i}=20$, $\Delta m_{i}=0.02{\bar{m}}_{i}\sin\left(t\right)$, ${\bar{I}}_{i}=10$, $\Delta I_{i}=0.02{\bar{I}}_{i}\sin\left(t\right)$, r=0.125, l=0.3, $r_{s}=0.9$, d=0.2, $K_{i}=I_{3\times 3}$, $D_{ai}=0.2I_{3\times 3}$, $\phi_{i}=0.8+0.2sign\left(sin\left(t\right)\right)$. The cyber interference input can be expressed as

$$\begin{array}{cc} \nu_{a1} & =\left[\begin{array}{c} sin(t)\\ cos(t)\\ sin(2t) \end{array}\right],\nu_{a2}=\left[\begin{array}{c} cos(t)\\ sin(t+1)\\ sin(3t) \end{array}\right],\nu_{a3}=\left[\begin{array}{c} sin(2t)\\ cos(1.5t)\\ sin(t) \end{array}\right],\\ \nu_{a4} & =\left[\begin{array}{c} cos(2t)\\ sin(2t)\\ sin(3t) \end{array}\right],\nu_{a5}=\left[\begin{array}{c} sin(t)\\ cos(t)\\ sin(0.5t) \end{array}\right],\nu_{a6}=\left[\begin{array}{c} cos(3t)\\ sin(t)\\ sin(1.5t) \end{array}\right]. \end{array}$$

Subject to Assumption 5, we choose $\delta_{E_{i}}=-0.02$,

(68) $$\begin{array}{ccc} \Theta_{i}\left(\gamma_{i},p^{i},{\dot{p}}^{i},t\right) & = & \gamma_{i1}∥p^{i}∥+\gamma_{i2}{∥p^{i}∥}^{2}\\ & & +\;\gamma_{i3}∥{\dot{p}}^{i}∥+\gamma_{i4}{∥{\dot{p}}^{i}∥}^{2}+\gamma_{i5}\\ & = & \gamma_{i}^{T}{\left[∥p^{i}∥,{∥p^{i}∥}^{2},∥{\dot{p}}^{i}∥,{∥{\dot{p}}^{i}∥}^{2},1\right]}^{T}, \end{array}$$

where $\gamma_{i1}$, $\gamma_{i2}$, $\gamma_{i3}$ $\gamma_{i4}$, $\gamma_{i5}$ are unknown and positive parameters, and $\gamma_{i}={\left[\gamma_{i1},\gamma_{i2},\gamma_{i3},\gamma_{i4},\gamma_{i5}\right]}^{T}$. The adaptive law is given as

(69) $$\begin{array}{c} \dot{\hat{\gamma_{i}}}=Y_{i1}{\left[∥p^{i}∥,{∥p^{i}∥}^{2},∥{\dot{p}}^{i}∥,{∥{\dot{p}}^{i}∥}^{2},1\right]}^{T}∥\epsilon_{i}∥-Y_{i2}{\hat{\gamma }}_{i}. \end{array}$$

The desired trajectory (target) which the swarm centroid need to follow is given as a ellipse:

(70) $$\left\{ \begin{array}{c} x^{*}-20\cos\left(0.1t\right)=0,\\ y^{*}-16\sin\left(0.1t\right)=0. \end{array}\right.$$

We choose the cooperative control parameters as $\kappa_{i}=0.5$, $Y_{i1}=10$, $Y_{i2}=1$, ${\hat{\gamma }}_{i}\left(0\right)={\left[0.1,0.002,0.1,0.1,0.1\right]}^{T}$. The initial conditions for each vehicle can be set as shown in Table 2.

**Table 2:** **Initial conditions for each vehicle**.

Notation/Unit	i=1	i=2	i=3	i=4	i=5	i=6
$x_{i}\left(0\right)/m$	16	18	20	20	22	24
$y_{i}\left(0\right)/m$	4	−2	2	−4	2	−3
${\dot{x}}_{i}\left(0\right)/\left(m/s\right)$	0	0	0	0	0	0
${\dot{y}}_{i}\left(0\right)/\left(m/s\right)$	0	0	0	0	0	0
$\varphi_{i}\left(0\right)/rad$	π/10	π/10	π/10	π/10	π/10	π/10
${\dot{\varphi }}_{i}\left(0\right)/\left(rad/s\right)$	0.02	0.02	0.02	0.02	0.02	0.02

It is noted that vehicles in the swarm system do not start with the constraint manifold when t=0. Based on the above settings, the simulation results are shown in Figs. 3–22.

**Fig. 3:**
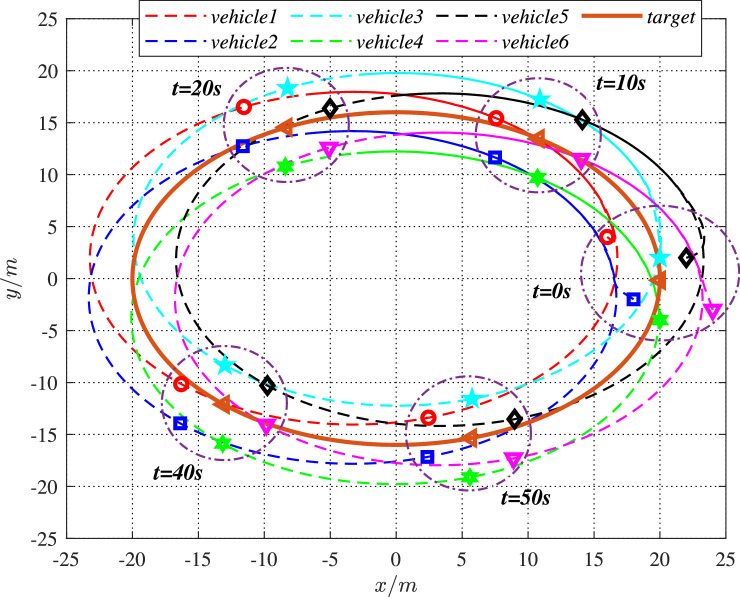
**The trajectories of the UGV swarm**.

**Fig. 4:**
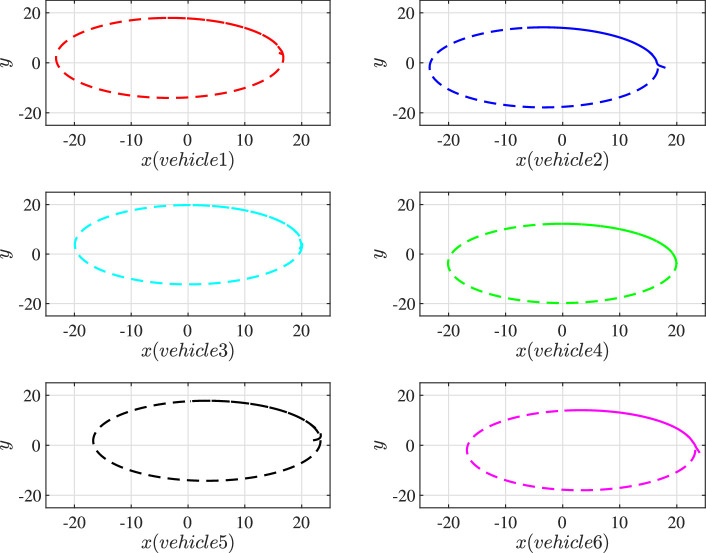
**Individual trajectories of 6 vehicles**.

Fig. 3 depicts the trajectories of the 6 vehicles in the swarm system under the cooperative control (31). For better distinguish, the trajectories, starting positions and ending positions of UGVs are identified by different color lines and markers. At the initial moment, the UGVs are arbitrarily distributed. Driven by the designed control, the UGV swarm suffering form cyber interference can move along the desired trajectory (the “target” line) and gradually form a stable formation. It can be seen that the target are always surrounded by the 6 vehicles, which shows the cooperative hunting performance of the swarm. In Fig. 4, the trajectories of these vehicles are multiple ellipses around the tracking target, which further demonstrates the tracking performance of the swarm in a clearer way.

**Fig. 5:**
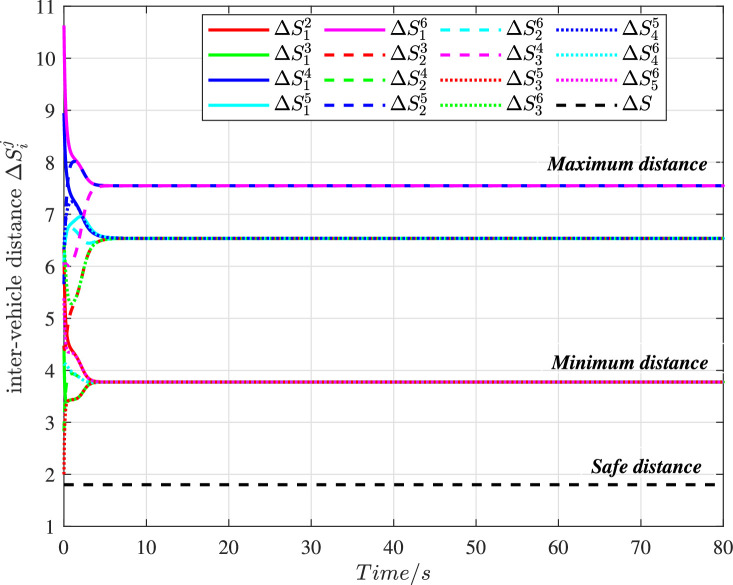
**The histories of inter-vehicle distance in the swarm system**.

We define $\Delta S_{i}^{j}=\sqrt{{\left(x_{i}-x_{j}\right)}^{2}+{\left(y_{i}-y_{j}\right)}^{2}}$ as the inter-vehicle distance to observe the position relationship of unmanned ground vehicles at different times, and the histories of which can be shown in Fig. 5. For the safe radius $r_{s}$, we have the safe distance $\Delta S=2r_{s}=1.8$. It can be seen that the distances $\Delta S_{i}^{j}$ are all significantly greater than the safe distance ΔS. That means, the collision avoidance of the swarm system can be guaranteed. Furthermore, these distances can quickly reach an equilibrium value and maintain this stable state, which further reflects that the swarm system under cyber interference can accomplish formation objectives under the proposed control. The maximum distance makes the formation more compact and further shows that the swarm size is bounded, which is consistent with Performance 2.

**Fig. 6:**
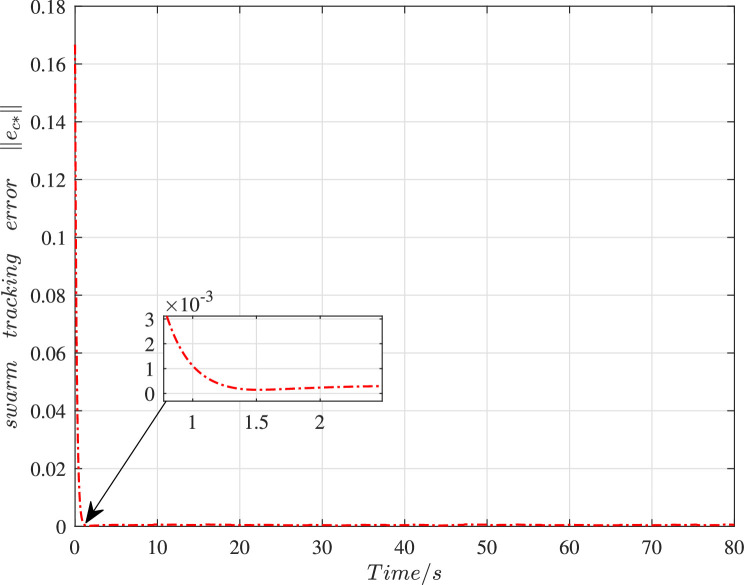
**The history of the swarm tracking error**$∥e_{c*}∥$.

**Fig. 7:**
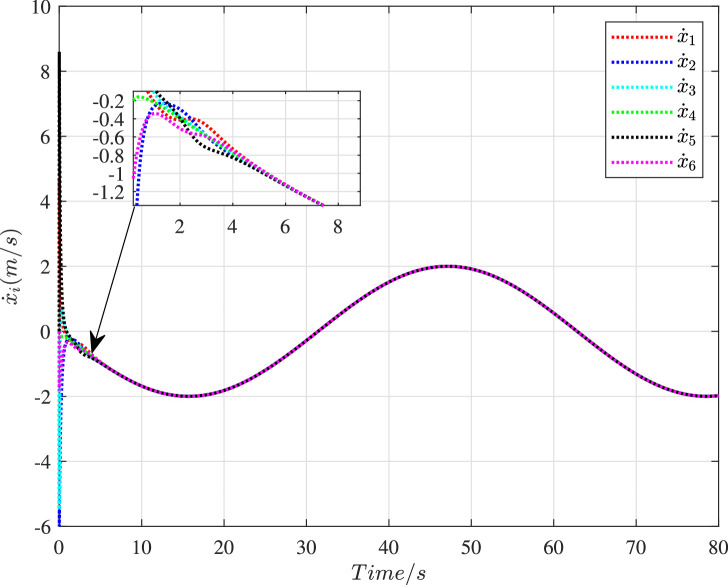
**The velocity histories of 6 vehicles in the X-direction**.

**Fig. 8:**
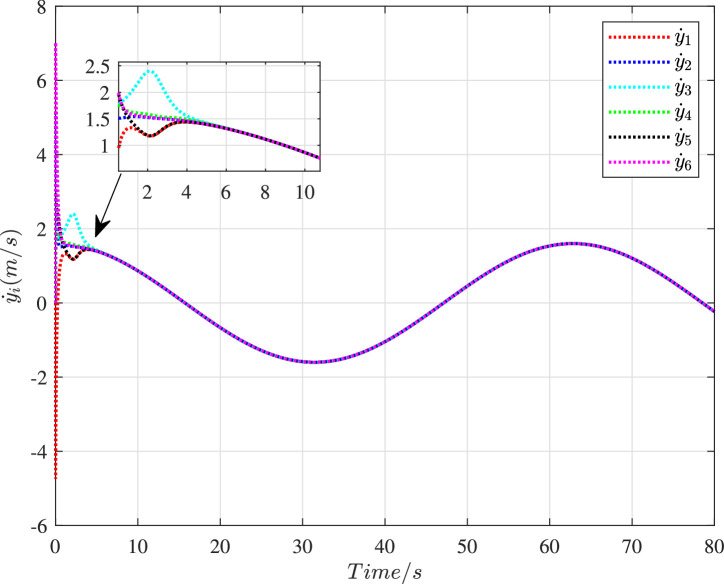
**The velocity histories of 6 vehicles in the Y-direction**.

Fig. 6 shows the time histories of the swarm tracking error under the cooperative control. Apparently, the tracking error $∥e_{c*}∥$ could settle to zero within 1.5 s. In Figs. 7 and 8, the histories of velocity in the X-direction and the Y-direction are presented respectively. It is obvious that the velocity state of these vehicles could realize the consensus after 4 s despite the existence of cyber interference.

**Fig. 9:**
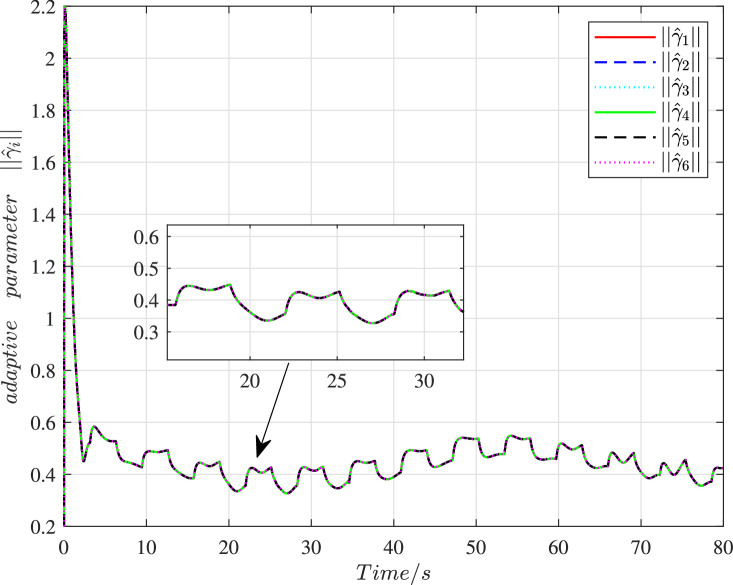
**Adaptive parameter**${\hat{\gamma }}_{i}$**history**.

**Fig. 10:**
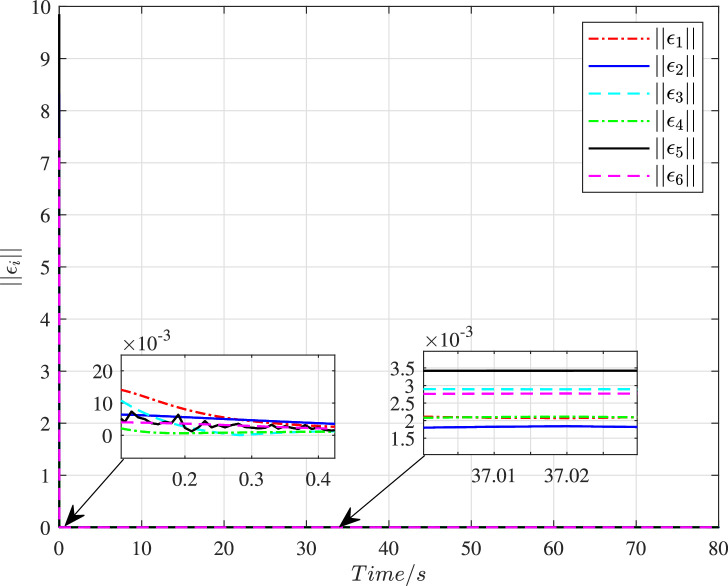
**History of the constraint following error**$∥\epsilon_{i}∥$.

**Fig. 11:**
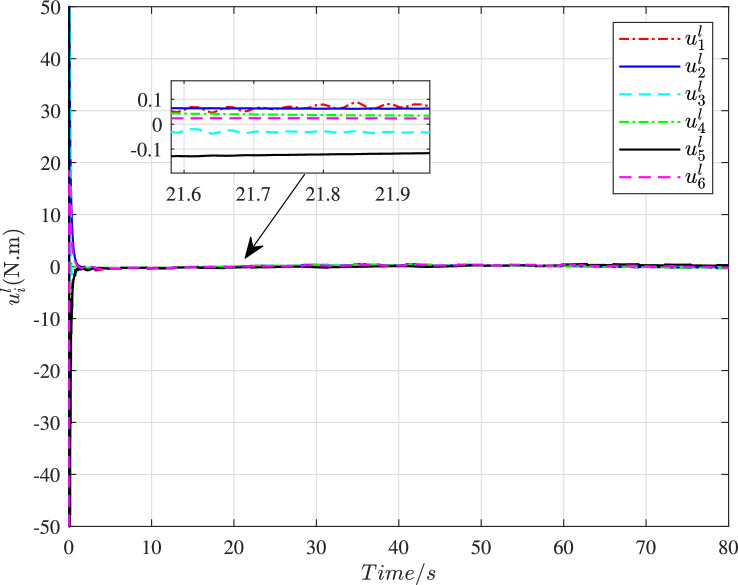
**The histories of the control torque**$u_{i}^{l}$**in the left wheel**.

**Fig. 12:**
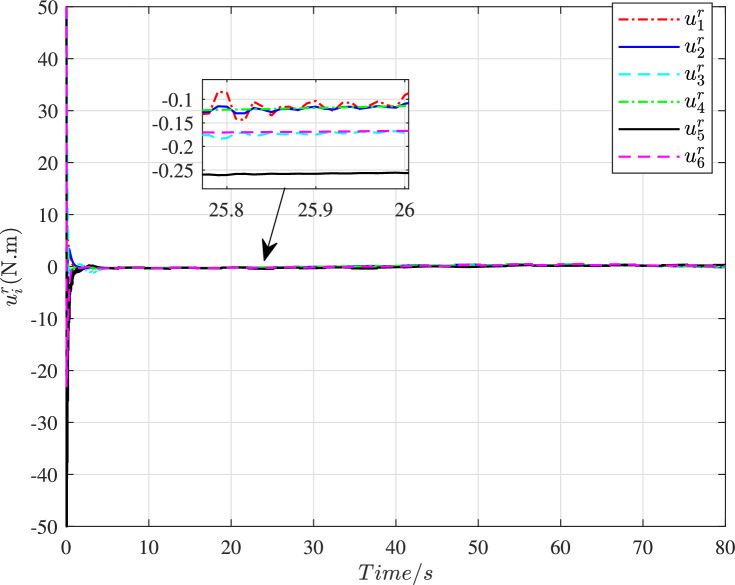
**The histories of the control torque**$u_{i}^{r}$**in the right wheel**.

The time histories of the adaptive parameters $∥{\hat{\gamma }}_{i}∥$ can be shown in Fig. 9. These adaptive parameters would first increase to the maximum value, then gradually decay, and ultimately change in a stable region. Fig. 10 depicts the time variation of the constraint following error $∥\epsilon_{i}∥$. It can be seen that these errors will quickly converge to zero even with a large initial deviation, so the constraint following performance under cyber interference can be sufficiently guaranteed. Figs. 11 and 12 demonstrate the control torques of driving wheels for all UGVs in the swarm system. They could gradually drop to a smaller region and their maximum value will not exceed the upper limit 50N.m.

**Fig. 13:**
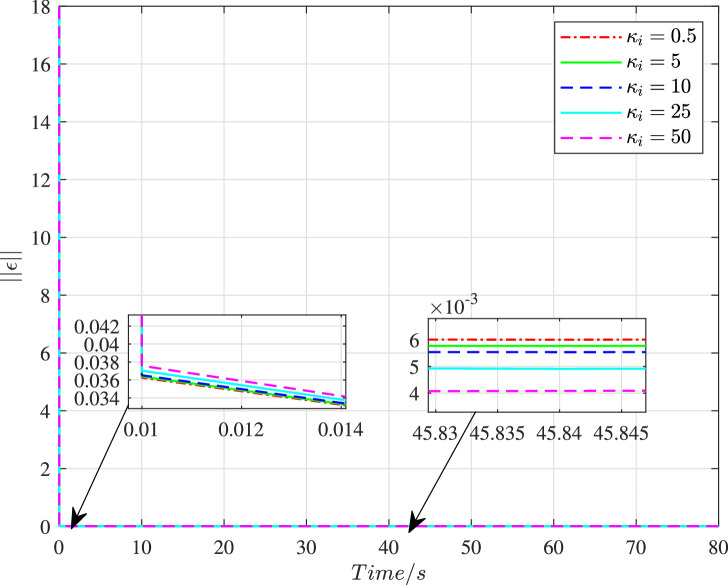
**The overall constraint following error**∥ϵ∥**with different**$\kappa_{i}$.

**Fig. 14:**
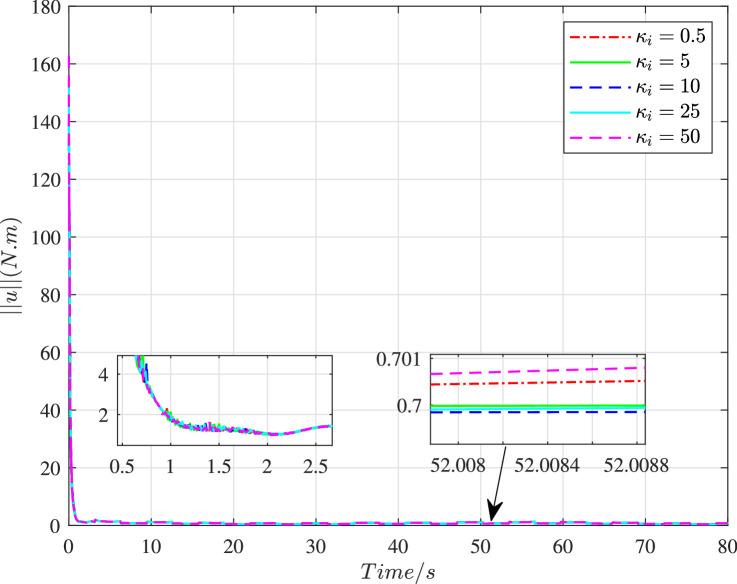
**The overall control cost**∥u∥**with different**$\kappa_{i}$.

**Fig. 15:**
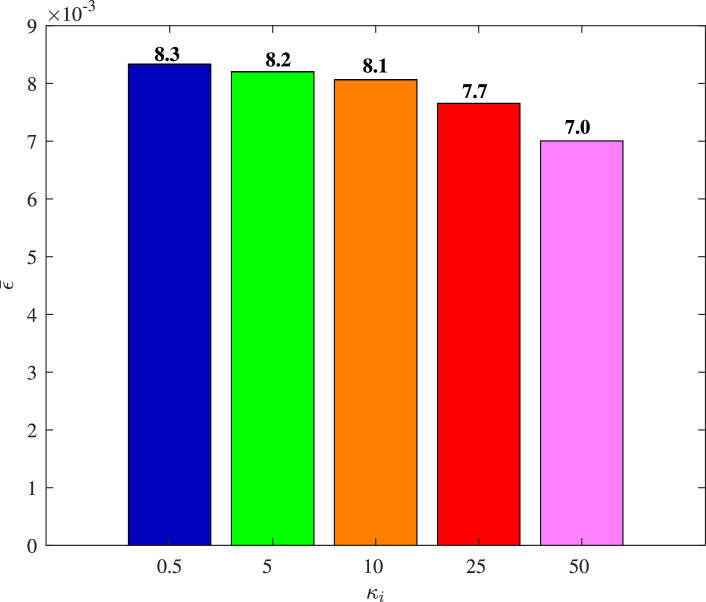
**The average constraint following error**$\bar{\epsilon }$**with different**$\kappa_{i}$.

**Fig. 16:**
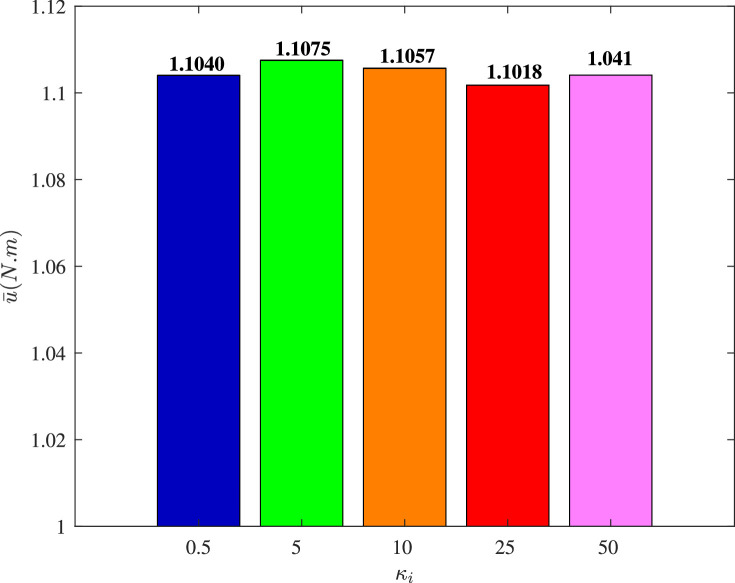
**The average control cost**$\bar{u}$**with different**$\kappa_{i}$.

**Fig. 17:**
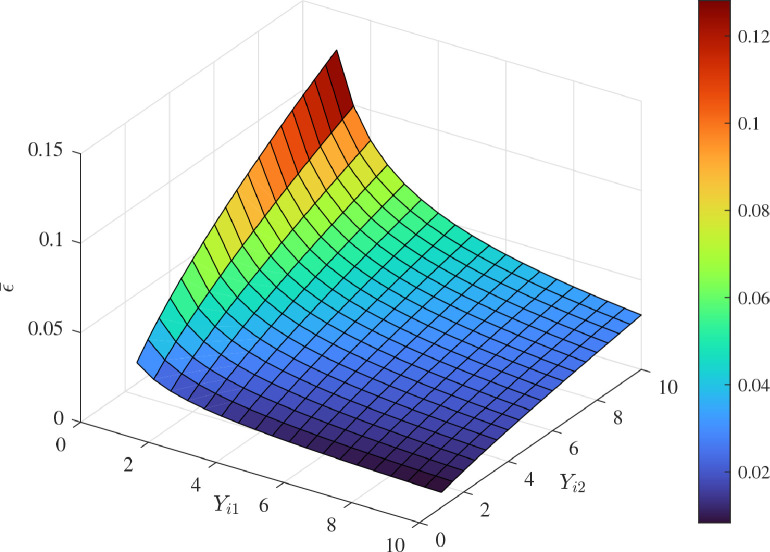
**The average constraint following error**$\bar{\epsilon }$**with different**$Y_{i1}$ and $Y_{i2}$.

**Fig. 18:**
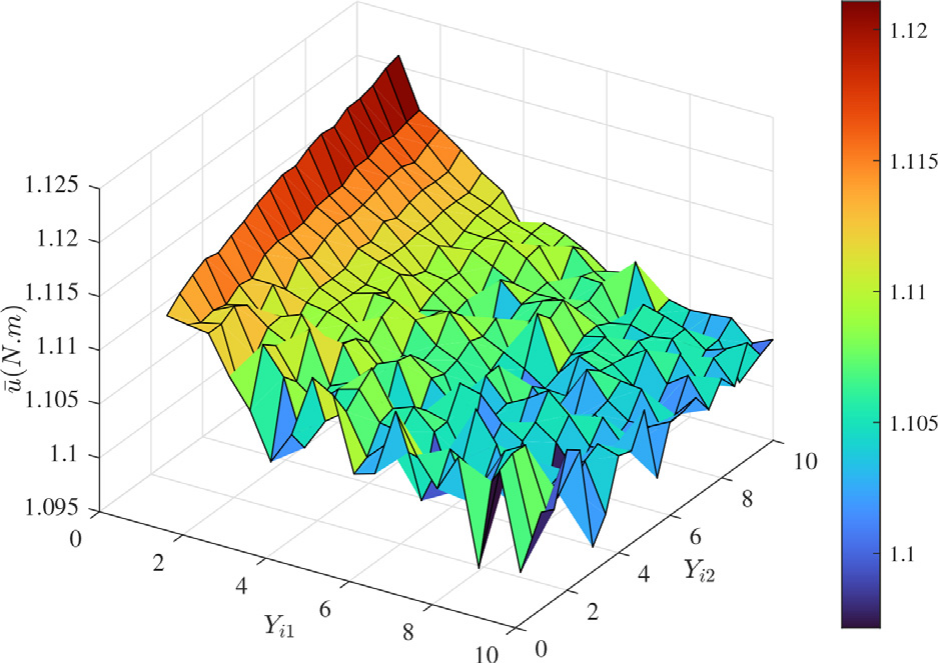
**The average control cost**$\bar{u}$**with different**$Y_{i1}$**and**$Y_{i2}$.

Figs. 13 and 14 show the time histories of overall constraint following error ∥ϵ∥ and the overall control cost ∥u∥ with different $\kappa_{i}$. These errors show the similar trends, so they could all quickly converge to zero. The control cost can all drop to a smaller region with different $\kappa_{i}$. To explore the influence of $\kappa_{i}$, Figs. 15 and 16 compare the average constraint following error $\bar{\epsilon }$ and the average control cost $\bar{u}$ with different $\kappa_{i}$. The greater $\kappa_{i}$ may reduce the average constraint following error but require different average control cost, which can serve as the reference for the choice of appropriate parameters. Figs. 17 and 18 compare the average constraint following error $\bar{\epsilon }$ and the average control cost $\bar{u}$ with different $Y_{i1}$ and $Y_{i2}$. The greater $Y_{i1}$ and smaller $Y_{i2}$ may lead to a smaller average constraint following error. The average control cost with different values of $Y_{i1}$ and $Y_{i2}$ is within a small range, and the average control cost is small when the value of $Y_{i1}$ is large and that of $Y_{i2}$ is small.

**Fig. 19:**
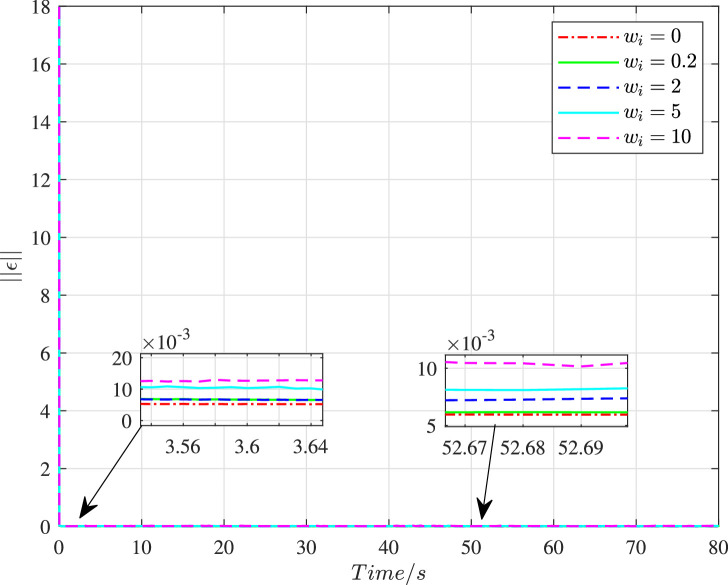
**The overall constraint following error**∥ϵ∥**with different**$w_{i}$.

**Fig. 20:**
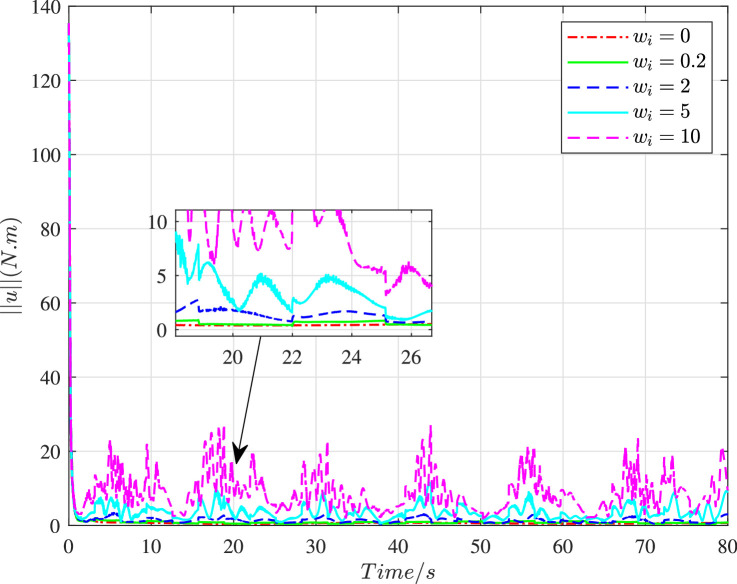
**The overall control cost**∥u∥**with different**$w_{i}$.

**Fig. 21:**
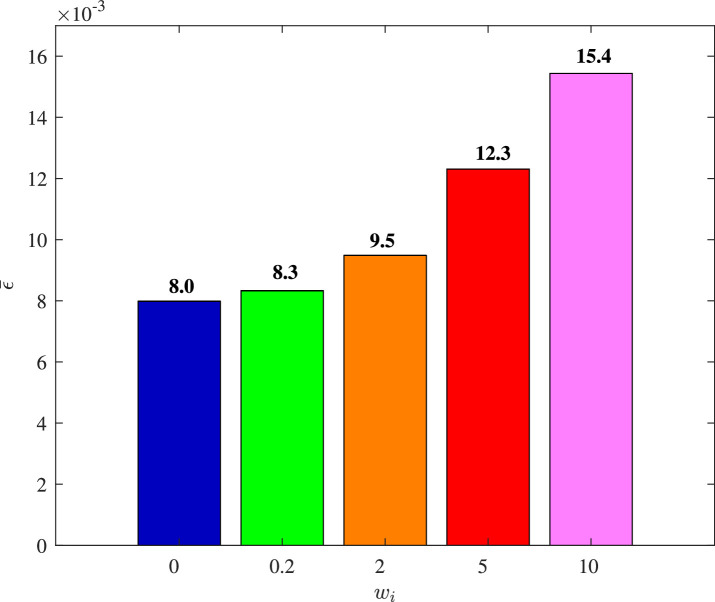
**The average constraint following error**$\bar{\epsilon }$**with different**$w_{i}$.

**Fig. 22:**
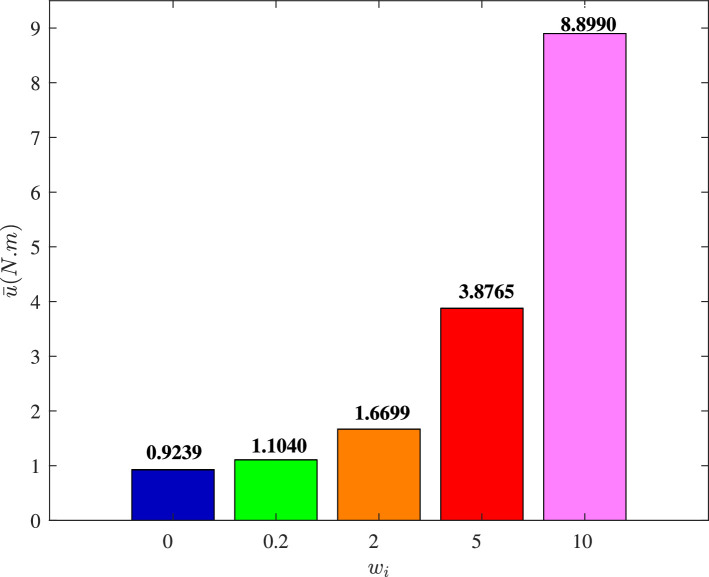
**The average control cost**$\bar{u}$**with different**$w_{i}$.

We set $D_{ai}=w_{i}I_{3\times 3}$, and choose different values of $w_{i}$ to illustrate the impact of cyber interference input with different intensities on the system performance, the simulation results of which are shown in Figs. 19–22. It is noted that $w_{i}=0$ means no cyber interference input, so $\phi_{i}=1$ meanwhile. The time histories of the overall constraint following error ∥ϵ∥ and the overall control cost ∥u∥ with various $w_{i}$ are shown in Figs. 19 and 20. It can be seen that the overall constraint following error can converge to a smaller range close to zero under the proposed control, whether there is cyber interference input with different intensities or no cyber interference input. Observing the overall constraint following error with different $w_{i}$, the error with no cyber interference input is smaller than that with cyber interference input, which is consistent with the performance analysis in Section 5. What’s more, the larger $w_{i}$ may require higher overall control cost. In order to intuitively explore the impact of cyber interference input, Figs. 21 and 22 compare the average constraint following error $\bar{\epsilon }$ and the average control cost $\bar{u}$ under different $w_{i}$. It is evident that the larger $w_{i}$, which means stronger cyber interference input, can result in greater average constraint following error and require higher average control cost.

## 7 Conclusion

The control issue of artificial swarm systems considering cyber interference is an inevitable topic. In face of challenges such as system dynamics, uncertainty and the effects of interference inputs, a skillful control approach is fundamental to the realization of the swarm performance. For the UGV swarm system, a cooperative control framework is elaborated, wherein various elements including constraints, kinematics, dynamics, uncertainty and cyber interference are taken into account. Inspired by the Udwadia-Kalaba approach, ideal kinematic model could be converted to the desirable constraints, then the nominal feedback control term and the adaptive robust control term can be designed accordingly. Under such a control framework, the global stability, uniform boundedness, uniform ultimate boundedness, compact formation, cooperative hunting and trajectory tracking can be guaranteed even there exist system uncertainty and cyber interference. The proposed cooperative control may have broad application potential such as military patrol, material allocation, target capture, etc.

## Declaration of competing interest

The authors declare that they have no conflicts of interest in this work.
